# Genome-wide identification and analysis of expression of pathogenesis-related protein 1 (PR-1) gene family in brown algae

**DOI:** 10.3389/fpls.2025.1754480

**Published:** 2026-01-23

**Authors:** Linhong Teng, Shuxia Liang, Jiayi Chen, Bostjan Kobe, Naihao Ye, Hui Wang, Jian Song

**Affiliations:** 1 College of Life Sciences, Dezhou University, Dezhou, China; 2 School of Chemistry and Molecular Biosciences, Institute for Molecular Bioscience and Australian Infectious Diseases Research Centre, University of Queensland, Brisbane, Queensland, Australia; 3 Yellow Sea Fisheries Research Institute, Chinese Academy of Fishery Sciences, Qingdao, China; 4 Qingdao Institute of Bioenergy and Bioprocess Technology Academy of Sciences, Shangdong Energy Institute, Qingdao, China

**Keywords:** brown algae, evolution, expression, gene family, PR-1

## Abstract

The pathogen-related protein 1 (PR-1) family plays an important role in plant response to biotic and abiotic stresses. PR-1 proteins have been studied in many plant species; however, they were not systematically studied in brown algae, which are important components of coastal ecosystems and have great economic value in the aquaculture industry. In the present study, we characterized the structure, evolution and expression of PR-1 proteins in brown algal genomes. A total of 141 PR-1s were identified in the 19 brown algal genomes, with an average of 7 genes in each species. Most PR-1s are acidic, while only 18 PR-1s are basic. Phylogenetic analysis showed that PR-1s in brown algae clustered into five clades, and showed no strong relationship with other lineages, suggesting an ancient origin. All the PR-1s contain a conserved CAP superfamily domain. Some PR-1s contain distinct functional domains, such as the WSC, Blect, and Bulb-type lectin domains, which are involved in carbohydrate binding. Their promoter regions were enriched in stress-response elements, hormone-response elements, growth and development elements. GO and KEGG annotation showed that brown algal PR-1 proteins may be involved in diverse roles and pathways. Moreover, expression analysis shows that some PR-1s, especially basic proteins are responsive to abiotic stress conditions and life stage development, further suggesting they participate in multiple functional pathways. Our results provide important data for future research on the function of brown algal PR-1 family genes.

## Introduction

Plants have developed complex mechanisms to fight against biotic and abiotic stresses. A key component are the pathogenesis-related (PR) genes, which are activated in response to pathogen infection and play diverse roles in the plant immune system (Zribi et al., 2021). PR proteins are classified into 17 families according to sequence similarities, enzyme properties and structural characteristics. Among them, the PR protein 1 (PR-1) family is a dominant group of PRs induced by pathogens or salicylic acid, and is commonly used as a marker for systemic acquired resistance (Breen et al., 2017). PR-1 family members were first identified in *Nicotiana tabacum*, where they accumulated rapidly during tobacco mosaic virus infection (Edreva, 2005). Since then, homologs of tobacco PR-1 proteins have been discovered in many plants, such as tomato, maize, rice and barley. Their homologs are also found in other plant lineages, as well as fungi, animals and human, although the biological activity of the PR-1 proteins is still unknown (Liu and Xue, 2006; Akbudak et al., 2020; Zhang et al., 2022). PR-1 proteins occur in multigene families and can be further classified as acidic or basic, according to their theoretical isoelectric point (pI). Numerous studies have demonstrated that PR-1 proteins accumulate abundantly in response to stress conditions, whereas the mode of action or relationship to other proteins is not yet clear (Breen et al., 2017). Tomato PR-1c inhibited the germination of oomycetes spores *in vitro*, and it reduced the diseased area of oomycete-infected leaves *in vivo* (Niderman et al., 1995). In grapevine, the basic-type VvPR1b1 gene offers high resistance against *Pseudomonas syringae* (Li et al., 2010). The PR-1 protein in *Wasabia japonica* exhibits strong inhibiting activity against fungi (Kiba et al., 2007). Despite that multiple evidence demonstrated the immune function of PR-1 proteins, researches revealed that only a few members of this family are induced and inhibit pathogens. For example, only one (At2g14610) and two genes in *Arabidopsis* and rice were found to be induced by the microbial pathogen *Pseudomonas syringae* or insects, respectively (Van Loon et al., 2006). The retention of the high number of PR-1s indicates an important function in plant life. There is also evidence that PR-1 proteins are involved in plant growth or development that is independent of stress responses (Hanfrey et al., 1996; Breen et al., 2017).

The “cysteine-rich secretory protein, antigen 5, and pathogenesis-related-1” (CAP) domain (IPR014044, PF00188) is a conserved domain shared by all PR-1 proteins. It has been found in a wide range of organisms, including bacteria, fungi, plants, and animals, with various roles in immune defence, cancer, reproduction, and morphogenesis (Breen et al., 2017). The CAP domain comprises approximately 150 amino acids and forms a conserved secondary structure composed of four alpha-helices and four beta-strands. It contains a caveolin-binding motif (CBM), which enables the sequestering of sterols from the membrane of pathogens (Schneiter and Di Pietro, 2013). The 11-residue peptide CAP-derived peptide (CAPE) located at the C-terminus of the CAP domain is reported to be responsible for triggering the PR-1 defense response (Chen et al., 2014). Such structures are responsible for their biological roles associated with defense to biotic and abiotic stress (Ghorbel et al., 2021).

Brown algae are the only multicellular organisms among the SAR supergroup (Stramenopiles, Alveolates, and Rhizarians), which originated from secondary endosymbiosis events (Keeling, 2010). Brown algae are important components of coastal ecosystems, often forming extensive underwater forests. Kelps, such as *Saccharina* and *Sargassum*, have great economic value in the aquaculture industry. However, the growth and production of brown seaweeds is often adversely affected by abiotic and biotic factors, especially pathogenic microorganisms. The molecular mechanisms of immunity have been largely studied in plants and animals, whereas the brown algal immunity mechanisms remain less characterized. Two classes of gene families, the NB-ARC-TPR genes and ROCO genes, were reported to play potential roles in recognition of the pathogens and the activation of innate immunity, and use exon shuffling and alternative splicing as mechanisms to generate a broad repertoire of immune responses in brown algae (Teng et al., 2023, Teng et al., 2024). Given the established significance of PR-1 proteins in pathogen defense, we aimed to perform a systematic analysis of brown algal PR-1 genes, by conducting a whole-genome comprehensive investigation. With the development of genome sequencing, the Phaeoexplorer project has provided an extensive genomic dataset of brown algae, spanning all the major orders of the Phaeophyceae (Denoeud et al, 2024). Using this data, the phylogeny and structure of the PR-1 genes were uncovered.

## Materials and methods

### Identification of PR-1 genes in brown algae

The genomes of brown algae were downloaded from the Phaeoexplorer project website https://phaeoexplorer.sb-roscoff.fr/ (Denoeud et al., 2024). Together with previously published brown algal genomes, 19 species with good genome quality were used to identify PR-1 genes. The HMM profile of PR-1 proteins SSF55797 was used to perform an hmmsearch in the brown algal proteomes. All output proteins were then verified using the online InterProscan program, to confirm the presence of CAP domain (IPR014044, PF00188).

### Sequence analysis

Theoretical isoelectric point (PI) and molecular weight (MW) were calculated using ProtParam (https://web.expasy.org/protparam/). Subcellular localization was predicted using Euk-mPLoc 2.0 (http://www.csbio.sjtu.edu.cn/bioinf/euk-multi-2/) (Chou and Shen, 2010). The protein transmembrane helices were predicted by DeepTMHMM (https://dtu.biolib.com/DeepTMHMM/) (Hallgren et al., 2022). Signal peptide were predicted using the SignalP5.0 server (https://services.healthtech.dtu.dk/service.php?SignalP-5.0) (Almagro Armenteros et al., 2019). Predicted phosphorylation sites of PR-1 proteins such as serine, threonine or tyrosine phosphorylation sites were identified using NetPhos 3.1 server (http://www.cbs.dtu.dk/services/NetPhos/) (Blom et al., 2004). The domain composition of PR-1 proteins was identified using InterProScan online. Intron and exon information of PR-1 genes was extracted from GFF files. The 3D structure was acquired in the online Alphafold Protein Structure Database https://alphafold.ebi.ac.uk/. Conserved motifs of PR-1 proteins were identified using MEME (http://meme-suite.org/tools/meme). The BetaCavityWeb server (http://voronoi.hanyang.ac.kr/betacavityweb) was used to predict the number of channel structures (Kim et al., 2015). The CASTp 3.0 (Computed Atlas of Surface Topography of proteins) online server (http://sts.bioe.uic.edu/castp/calculation.html) was used to predict the active site pockets of the EsPR-1 protein (Tian et al., 2018). Intrinsic disordered regions of PR-1 proteins were predicted using PrDOS website (https://prdos.hgc.jp/cgi-bin/top.cgi) (Ishida and Kinoshita, 2007). Sequence logos of CAP domain were generated using online weblogo3 (https://weblogo.threeplusone.com/create.cgi) (Crooks et al., 2004). Cis-elements analysis: Sequences of genomic fragments positioned 2-kb upstream from the start codon of PR-1 gene were extracted from the brown algal genomes, and then cis-elements were predicted using PlantCARE. (http://bioinformatics.psb.ugent.be/webtools/plantcare/html/) (Lescot et al., 2002).

### Phylogenetic analysis

The CAP domains of a total of 141 PR-1 proteins in brown algae were extracted to construct the phylogenetic tree. The CAP domains were aligned using MUSCLE V5 (Edgar, 2022), then the ML tree was constructed using the RaxML-NG program with WAG+G4 model predicted by ModelTest-NG (Kozlov et al., 2019). Bootstrapping with 1000 resamplings was performed to obtain the confidence support value. To trace the origin of brown algal PR-1 proteins in a broader context, the phylogenetic tree including more organisms was constructed. Firstly, the HMM profile of brown algal CAP domain was built using hmmbuild. Then, this HMM profile was used to perform an hmmsearch in the NR database. After deleting the redundant sequences using CD-hit, the resulting proteins were manually checked using the InterProscan, to confirm the presence of CAP domains. Then, the CAP domains of acquired sequences were extracted and used to construct the big phylogenetic tree.

### Expression of PR-1 genes in brown algae

The expression patterns of PR-1 genes under different abiotic stresses and life-cycle stages were examined using the available transcriptome data. The RNA-seq data of female gametophytes, male gametophytes, their eggs and sperms, zygotes, embryo, and mature sporophytes were used to compare the expression of genes between different developmental stages (Lipinska et al., 2019; Denoeud et al., 2024). The expression levels between the freshwater and seawater species of *Pleurocladia lacustris* and *Porterinema fluviatile* were compared. Furthermore, previous microarray data of the *Ectocarpus* transcriptome (Dittami et al., 2009; Ritter et al., 2014) were used to explore the expression changes of PR-1 genes in response to abiotic stresses, including copper stress, hyposaline stress, hypersaline stress, and oxidative stress. The stress responses of PR-1 in *S. japonica* under high light, high temperature, acidification, hyposaline and hypersaline conditions were explored using digital gene expression (DGE) library sequencing (Zhang et al., 2021). Genes with a P-value < 0.05 and a log2 (fold change) >1 were considered as significantly differentially expressed genes. Hierarchical cluster heatmaps were created using the R package.

## Results

A total of 141 PR-1 proteins were identified in 19 brown algal genomes. The number in each brown alga ranges from 1 to 20, with an average of 7 genes in each species (**Table 1**). The protein lengths span 136 to 1994 amino acids, with the molecular weights ranging from 14.9 to 215.2 kDa. With respect to their isoelectric point values, most of the PR-1s are acidic, and only about 12% are basic. Most PR-1s were predicted to localize in extracellular spaces, while 13 PR-1s in the cytoplasm or the nucleus. 42 PR-1s harbored a signal peptide at their N-terminus. Most sequences do not have transmembrane helixes. The number of the putative phosphorylation sites in the PR-1 proteins ranged from 8 to 473. Details about the properties of these PR-1 proteins are presented in **Supplementary Table S1**.

**Table 1:** The identified PR-1 protein numbers in brown algae.

Brown algae	PR-1 number	Acidic	Basic
*Chordaria linearis*	5	4	1
*Cladosiphon okamuranus*	10	8	2
*Desmarestia herbacea*	4	3	1
*Dictyota dichotoma*	11	10	1
*Ectocarpus crouaniorum*	7	6	1
*Ectocarpus fasciculatus*	3	3	0
*Ectocarpus siliculosus*	8	7	1
*Fucus serratus*	6	5	1
*Heterosigma akashiwo*	1	1	0
*Sargassum fusiforme*	20	19	1
*Pleurocladia lacustris*	5	4	1
*Porterinema fluviatile*	3	3	0
*Pylaiella littoralis*	6	6	0
*Saccharina latissima*	5	4	1
*Schizocladia ischiensis*	3	2	1
*Scytosiphon promiscuus*	11	9	2
*Saccharina japonica*	13	12	1
*Ectocarpus* sp.	7	6	1
*Nemacystus decipiens*	13	11	2

### Phylogenetic analysis

**Figure 1:**
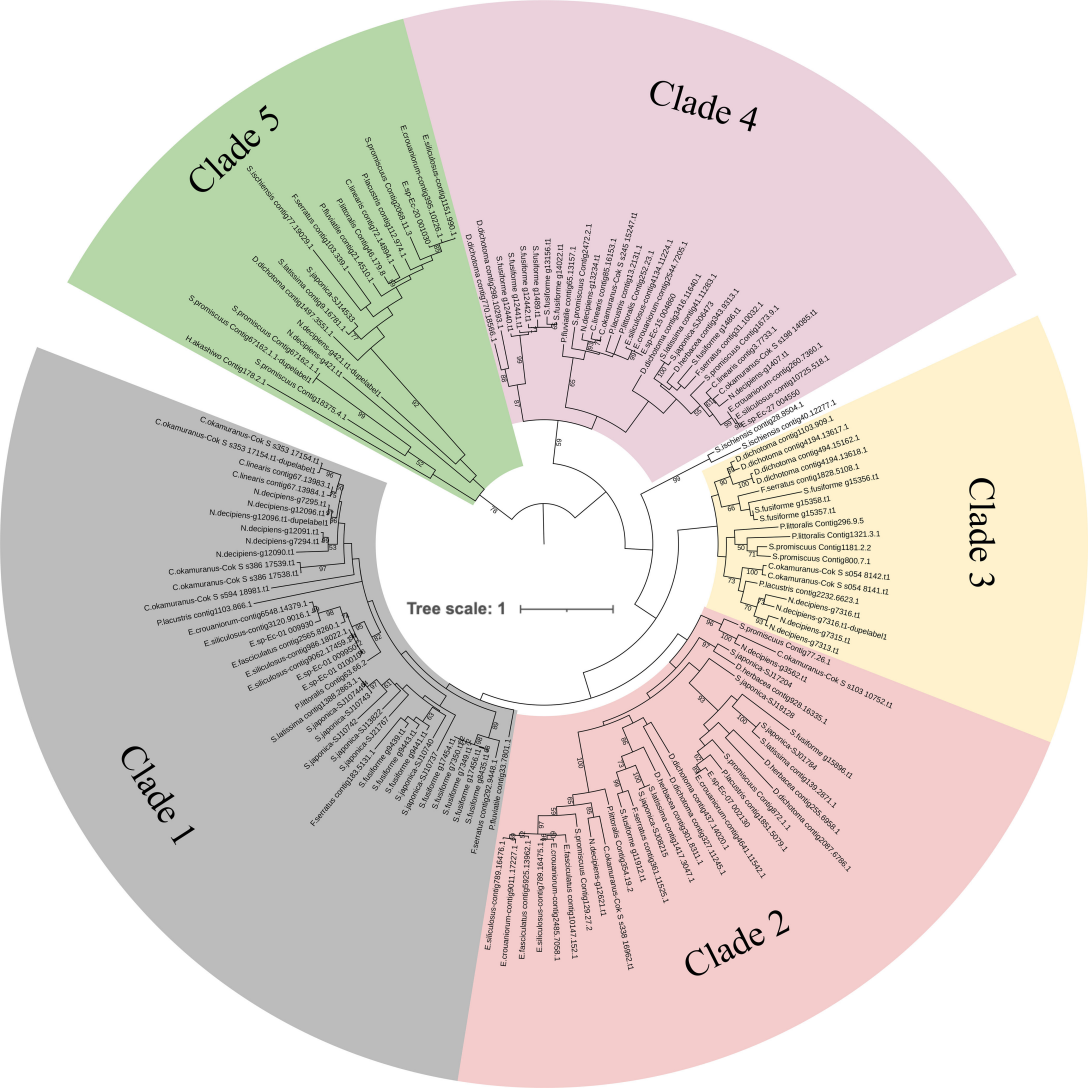
Phylogenetic tree of the brown algal PR-1 proteins. The ML tree was generated using RaxML-NG with the WAG+G4 model predicted by ModelTest-NG. Numbers on the nodes represent the bootstrap values larger than 50%.

In the tree built using the aligned CAP domains of the PR-1 proteins in brown algae, the 141 proteins clustered into five clades (**Figure 1**). Clade 1 contains 42 sequences from 14 species. Tandem duplication frequently occurred in this clade. *Sargassum fusiforme* has eight tandem duplicated members. *Nemacystus decipiens* and *Cladosiphon okamuranus* have a tandem-duplicated CAP domain within one gene. Different from tandem duplication in clade 1, clade 2 members are mainly composed of orthologous genes. The 32 genes in clade 2 formed five orthologous groups, and each group contained homologous genes from different species, suggesting that each group has originated from the common ancestor of brown algae. The 19 leaves from clade 3 came from eight species, which also belong to eight genera. Most PR-1s in clade 3 occurred from tandem duplication and PR-1s of each species clustered together, suggesting this clade existed in the common ancestor of brown algae, and remained and duplicated in some species after speciation, while they were lost in some genera, such as *Saccharina* and *Ectocarpus*. The status of clade 4 is similar to clade 2. It contains 31 genes from three orthologous groups, suggesting they are inherited from common ancestors of brown algae. Notably, one group in clade 4 exclusively contains basic PR-1s, suggestive of coevolutionary tracks of the proteins with similar isoelectric points. The 19 sequences in clade 5 seems to be distant from other clades. Indeed, when we explore the sequence characteristics, the genes of clade 5 showed distinct sequence and motif composition, suggesting its different nature from other PR-1 proteins.

**Figure 2:**
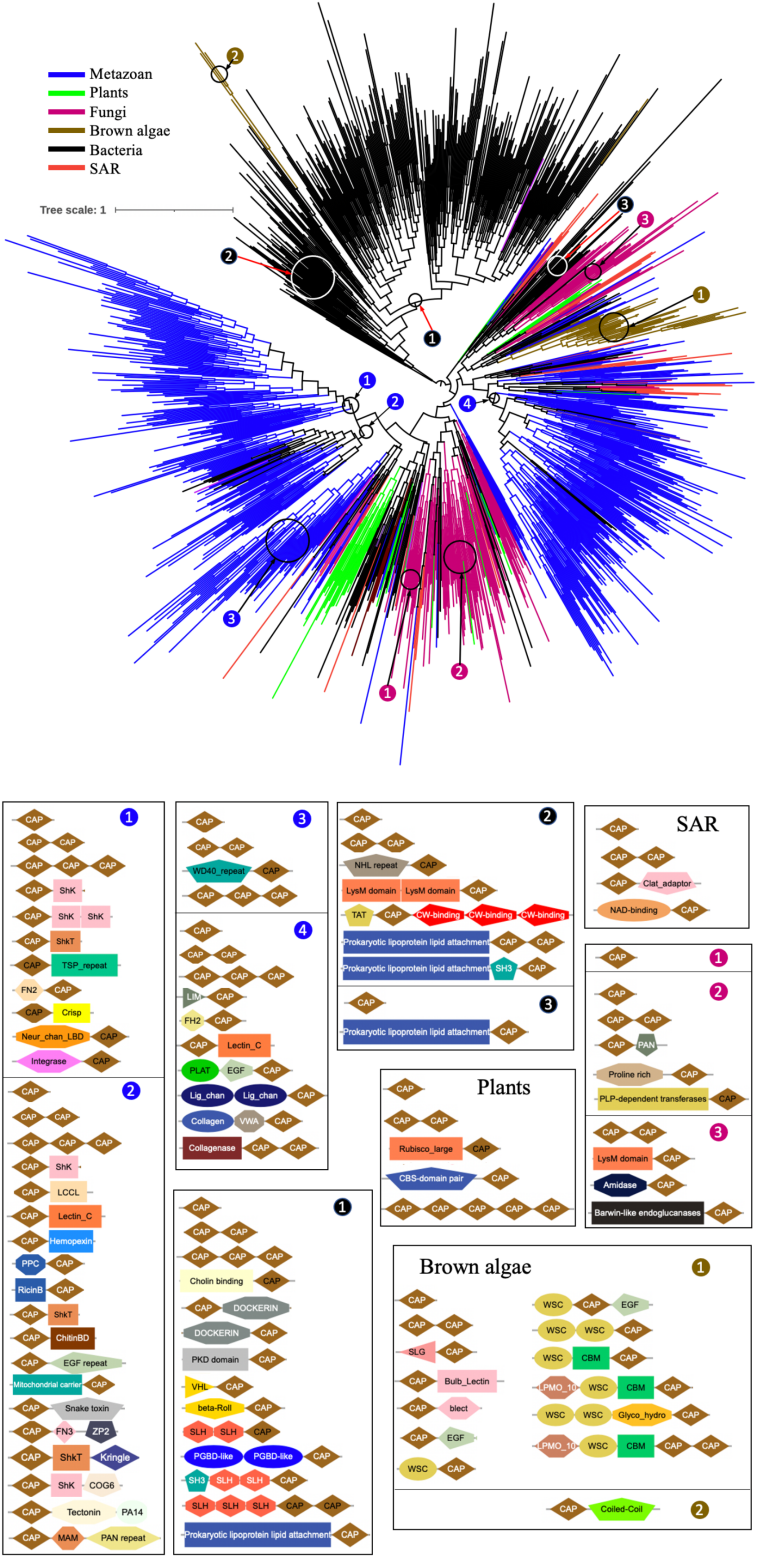
Phylogenetic tree and domain analysis of PR-1 sequences across a wide range of kingdoms. A total of 1228 CAP domain sequences were aligned and the ML tree was generated. Branch colors represent different kingdoms of life. Colored number on the tree denote the branches of the corresponding colored lineages. The domain combinations in each kingdom are shown below the tree.

To explore the origin of brown algal PR-1 proteins in a broader context, phylogenetic trees including more organisms were constructed. Firstly, we generated a hidden Markov model using the CAP domains of brown algal PR-1s. Using the downloaded 468,594,541 protein sequences in the NR database of NCBI, we performed an hmmsearch using the HMM profile of brown algal CAP domains in the PR-1 proteins, with the sequence reporting threshold of 1e-10. A total of 45,598 sequences were obtained. They were clustered using CD-hit with an identity threshold of 0.4, resulting in 4003 sequences. The CAP domains of these sequences were extracted and clustered again using the CD-hit with the threshold of 0.4, resulting in 1177 sequences. Meanwhile, the CAP domain of brown algal PR-1s were clustered using CD-hit with the threshold of 0.6, resulting in 51 sequences. Finally, a total of 1228 sequences were aligned and the ML tree was constructed (**Figure 2**). In this tree, using more representative sequences from the NR database, most CAP domains are from metazoans, and most of the CAP domain containing proteins in metazoans are cysteine-rich secretory proteins (CRISPs) and glioma GliPR1-like proteins, indicating that brown algal PR-1s have higher similarity with these genes. The four clades of brown algae, from clade 1 to clade 4, clustered together, while they showed no obvious affinity with other phyla. They are even distant with other SAR species, suggesting the PR-1s in brown algae evolved independently. Moreover, the members of clade 5, which is distant from the other four clades, are distributed in the bacteria group, confirming that clade 5 has a different origin from clades 1-4.

We further performed domain analysis across the phylogenetic tree. For sequences from all phyla, most proteins have one single CAP domain (IPR014044). However, examples of two or three tandem CAPs were also found. Diverse domains were identified to accompany the CAP domain in proteins from different organisms. In metazoans, there are at least 27 different domain combinations among the 517 sequences. The four subgroups of metazoan contain different domains. For bacteria, at least 22 different domain combinations were found in the 450 sequences, distributed in the three subgroups. These diverse domains indicate that these CAP proteins have developed various biological functions during evolution. Compared with the diverse domain composition in metazoans and bacteria, the numbers of sequences and domain types are much lower in fungi, plants and some SAR species, with 8, 5 and 4 identified domain compositions in 140, 24, and 28 sequences, respectively, indicating the conserved function in these lineages. Compared with other phyla, brown algal PR-1 proteins contain some distinct functional domains, such as the WSC domain (PS51212, IPR002889), the blect domain (IPR001480, SM00108), the bulb-type lectin domain (IPR001480), and the CBM domain (PS51164). The clade 5, which is distant from the other four clades, contains a long coiled-coil domain. Many brown algal PR-1s contain the WSC domain, which is a putative carbohydrate binding domain and contains up to eight conserved cysteine residues. SLG_2 (SM00321, IPR002889) is integrated into the WSC domain, and present in WSC proteins. The blect domain is integrated into the bulb-type lectin domain, which generally binds mannose. CBM is a carbohydrate-binding domain. These domains are specific to brown algal PR-1s and are not found in the CAP-containing proteins of other lineages.

### Sequence analysis

**Figure 3:**
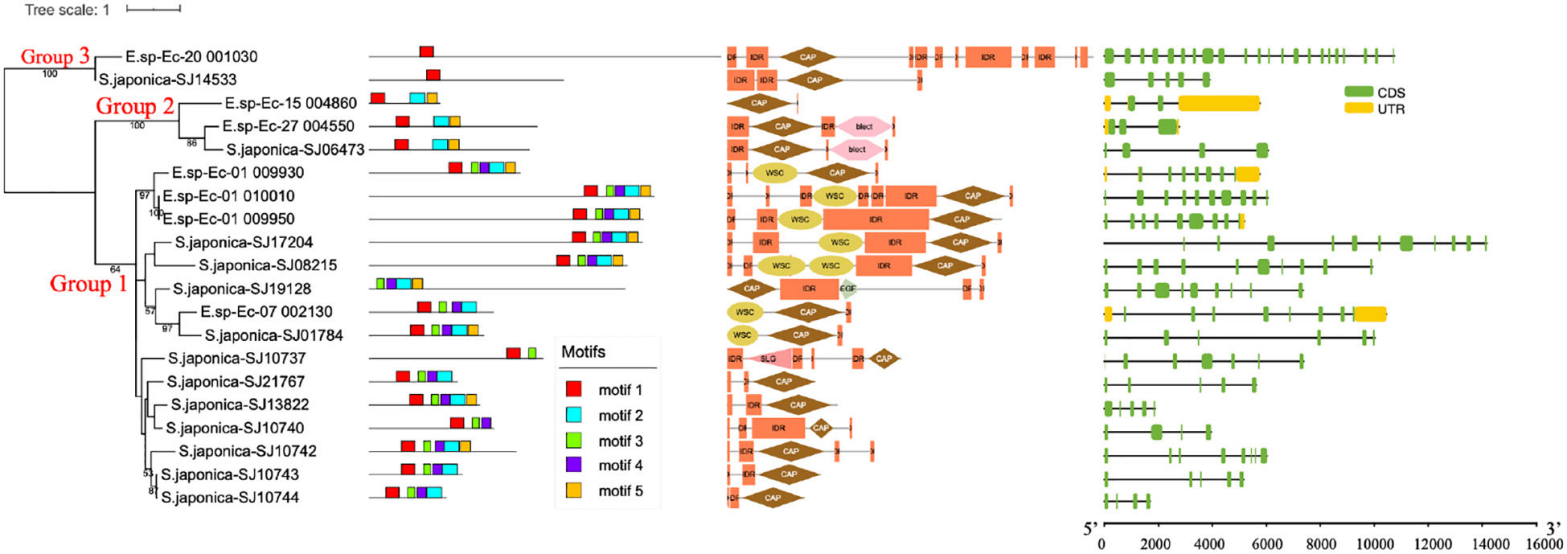
Phylogenetic tree of the PR-1 proteins in *Ectocarpus* and *S. japonica*. Conserved motifs, domain architecture and exon-intron structures are shown next to the tree.

**Figure 4:**
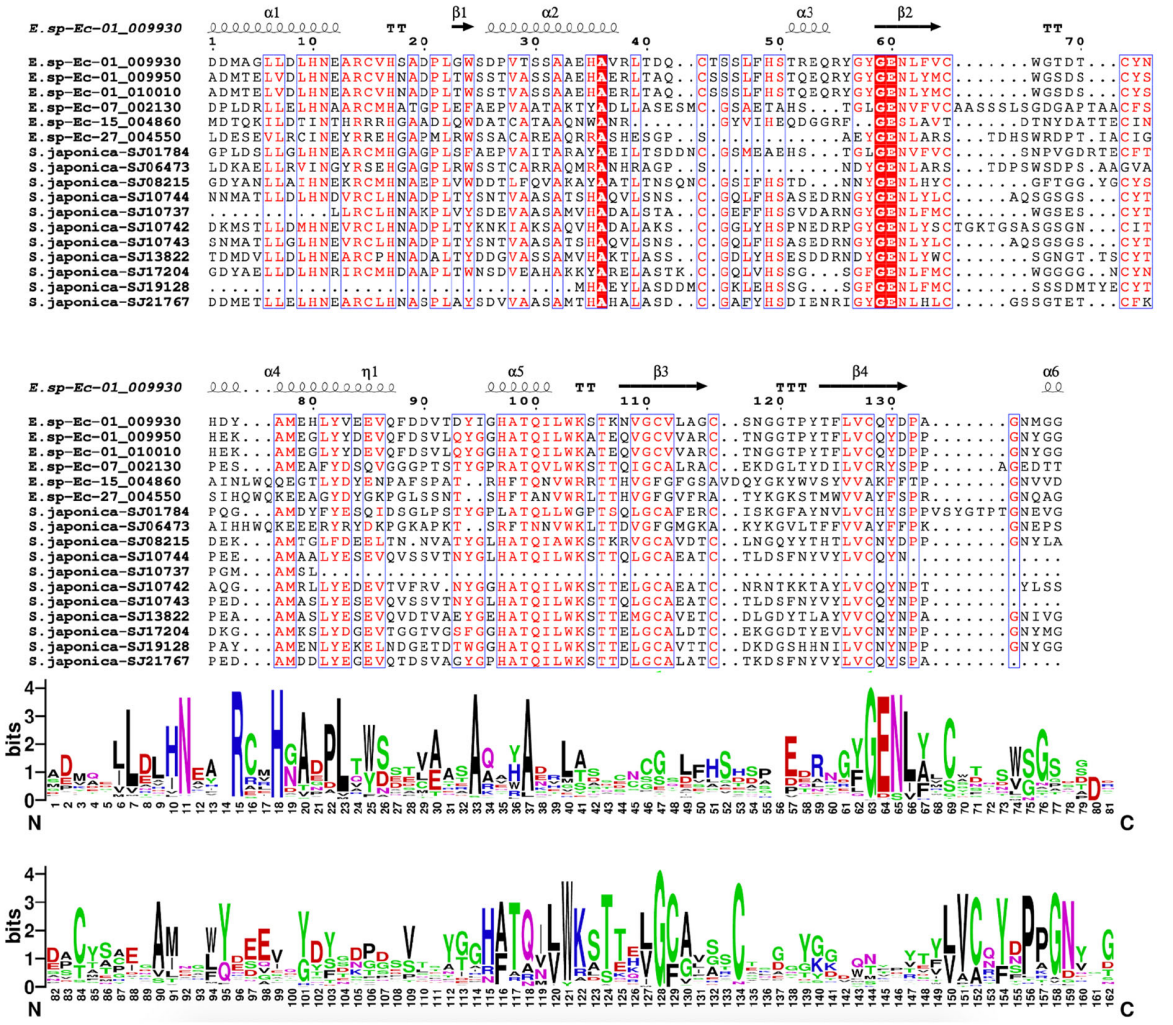
Multiple sequence alignments and sequence logo of the CAP domain of the EsPR-1 and SjPR-1 proteins.

We analyzed in detail the PR-1 sequence structures of the two representative brown algae, *Ectocarpus* sp. and *S. japonica* (**Figure 3**). The PR-1s of these two species formed three groups. Group 1 contains the genes from clade 1 and clade 2 in the tree shown in **Figure 1**. Group 2 and group 3 belong to clade 4 and clade 5, respectively. We used MEME to predict five conserved motifs. Most members in group 1 contain all five motifs. Group 2 contains motifs 1, 2 and 5, while group 3 contains only motif 1. Motif 5 corresponds to the C-terminal peptide (CAPE) of the CAP domain; it is involved in plant immune signalling and facilitates defence responses against microbial pathogens. The presence of motif 5 in groups 1 and 2 suggests their potential roles in immunity. The alignment and sequence logos of the CAP domains of groups 1 and 2 showed conserved secondary structure and six conserved cysteine residues (**Figure 4**). However, the presence of only motif 1 in group 3 suggests a distinct sequence. From the sequence logo of CAP domains, we further found that CAP domains of clade 5 exhibit a different amino acid composition from clades 1-4. Only two conserved cysteines were detected in clade 5, and no the conserved CAPE (PXGNXXG) was detected at the C-terminus of the CAP domain. We further checked the CAP domains in other lineages (**Supplementary Figure S1**). Despite the diverse amino acid composition of CAP domains, we found that the N-terminal LXXXNXXR sequence is a particularly well-conserved region throughout all the lineages, suggestive of an important functional role of this motif. The C-terminal CAPE (PXGNXXG) is conserved in brown algal clades 1-4, metazoan, fungi, and plants, while absent in clade 5 and bacterial CAP domains, further supporting the close relationship of clade 5 with bacteria, as found in the phylogenetic tree. Considering the role of CAPE in defence responses against microbial pathogens, its absence in bacteria seems understandable. Six conserved Cys residues were identified in clades 1–4 and plants. However, three and four Cys residues were identified in metazoans and fungi, respectively, and consequently, these proteins have fewer disulfide bridges than the plant ones. Notably, only two Cys residues were detected in clade 5, and no conserved Cys was found in bacteria, further suggesting their distinct characteristics, compared to other CAP domains.

With regards to the domain composition, some of the group 1 proteins have a WSC domain, while the group 2 proteins have the blect domain. Both the blect and WSC domains are carbohydrate-binding domains. Beside these functional domains, the long extended region of the proteins contains intrinsically disordered regions (IDRs), with the percentage ranging from 10% to 45% of each sequence. IDRs are reported to be involved in key cellular processes due to their conformational flexibility (Pazos et al., 2013). The high percentage of IDRs in the PR-1 proteins suggests their potential roles in cell regulation processes. The exon numbers of the PR-1 genes were found to range from two to 22, with an average number of seven in the two brown algal species. Generally, the exon number is higher than in plants.

### Cis-regulatory elements analysis

**Figure 5:**
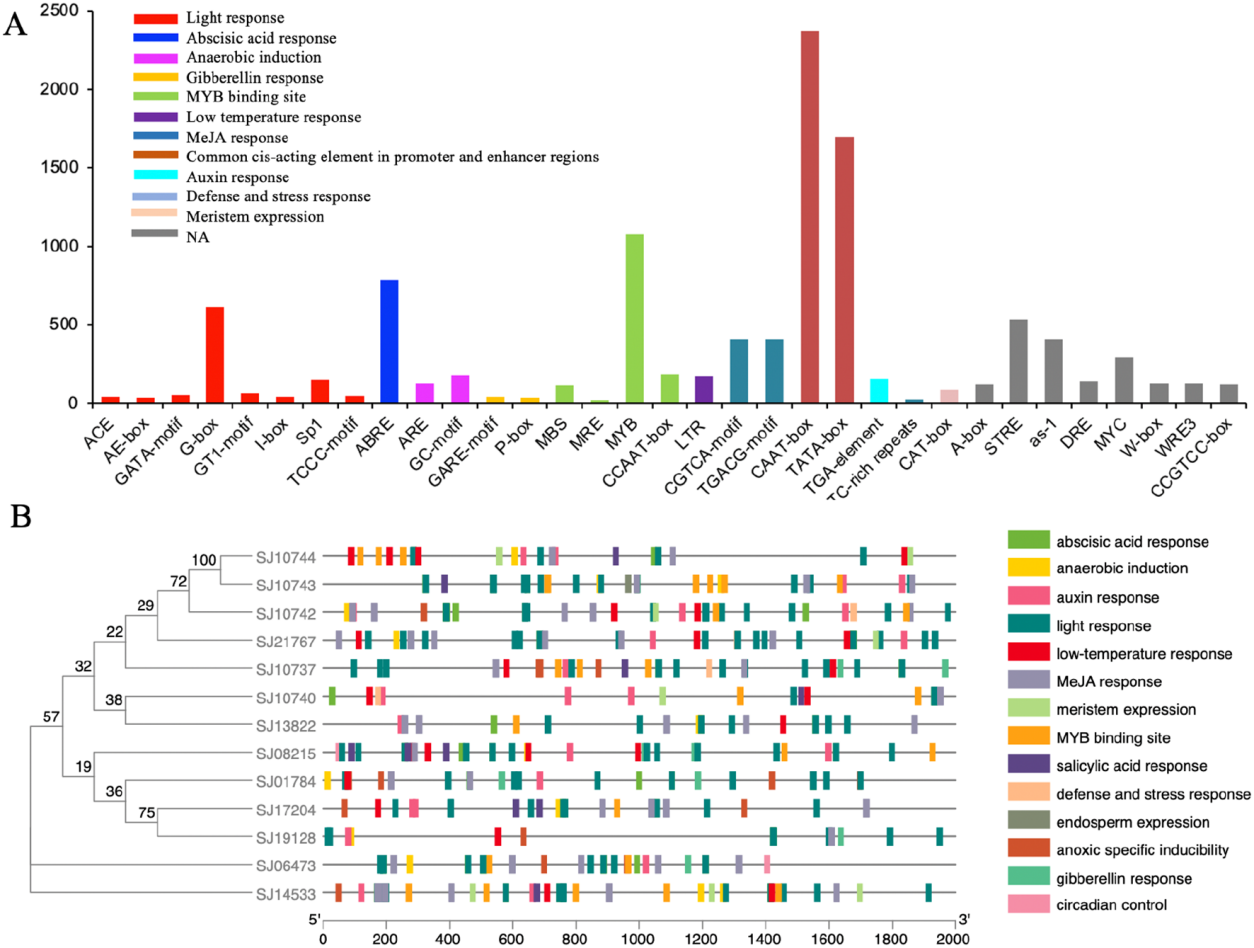
Analysis of cis-elements identified in the PR-1 promoter regions in brown algae. **(A)** the total number and classification of cis-elements of all the brown algal PR-1 genes. **(B)** The distribution of the cis-elements in the SjPR-1 promoters, determined using the TBtools software.

The cis-elements in the PR-1 promoters were analyzed using PlantCARE. The cis-elements are distributed over the 2.0 kb upstream promoter region. The cis-element number ranges from 11 to 200, with an average of 135 cis-elements per gene. Only 11 cis-elements were found in contig9062.17459.1 of *Ectocarpus siliculosus*, while the highest number of cis-elements was found in g9439.1 of *Sargassum fusiforme* (200), followed by contig77.19029.1 of *Schizocladia ischiensis* (194) and SJ10743 of *Saccharina japonica* (190). The results show many stress-response elements, hormone-response elements, growth and development elements, and transcriptional factor binding elements in the promoters. The most abundant cis-elements (CAAT-box, TATA-box) belong to the common cis-elements in promoter and enhancer regions, followed by cis-elements involved in MYB binding (MYB), abscisic acid response (ABRE), and light response (G-box). Various hormone response elements, responding to hormones such as abscisic acid (ABRE) and gibberellin (GARE-motif, P-box), MeJA (CGTCA-motif, TGACG-motif) and auxin (TGA-element) are present. These hormones are especially closely related to the abiotic or biotic stress resistance. In addition, cis elements in response to light (ACE, AE-box, I-box, G-box, GT1-motif, GATA-motif, Sp1, TCCC-motif), low temperature (LTR), drought (MBS), anaerobic stress (ARE, GC-motif), development (CAT-box), MYB binding site (MBS, MRE, MYB, CCAAT-box), defense and stress response (TC-rich repeat) are found to be widely distributed across the PR-1 promoter regions. When analyzing *S. japonica* in detail, a total of 1876 cis-elements were found in 13 genes of S. japonica. The most cis-elements were found in SJ10743 (190), followed by SJ10742 (184). As many as 19 kinds of light response elements were identified in SjPR-1 promoters, among them the G-box was present in all the 13 SjPR-1 gene promoters. As one of the important transcription factor families, MYB regulates many critical processes in growth and development. The binding domains of MYB were identified in all the 13 SjPR-1 genes, with a maximum number of 20 in SJ10743 and SJ14533, followed by SJ10742, SJ10737, SJ10740, and SJ08215, which have more than ten MYB binding sites, suggesting their potential roles regulated by MYB (**Figure 5**; **Supplementary Table S2**). However, PlantCARE was trained mainly on terrestrial plant promoters. Brown algae are phylogenetically distant from plants; using this tool to predict the cis-elements in brown algae may be not applicable, and need further confirmation by experiments.

### 3D structures of the EsPR-1 proteins

**Figure 6:**
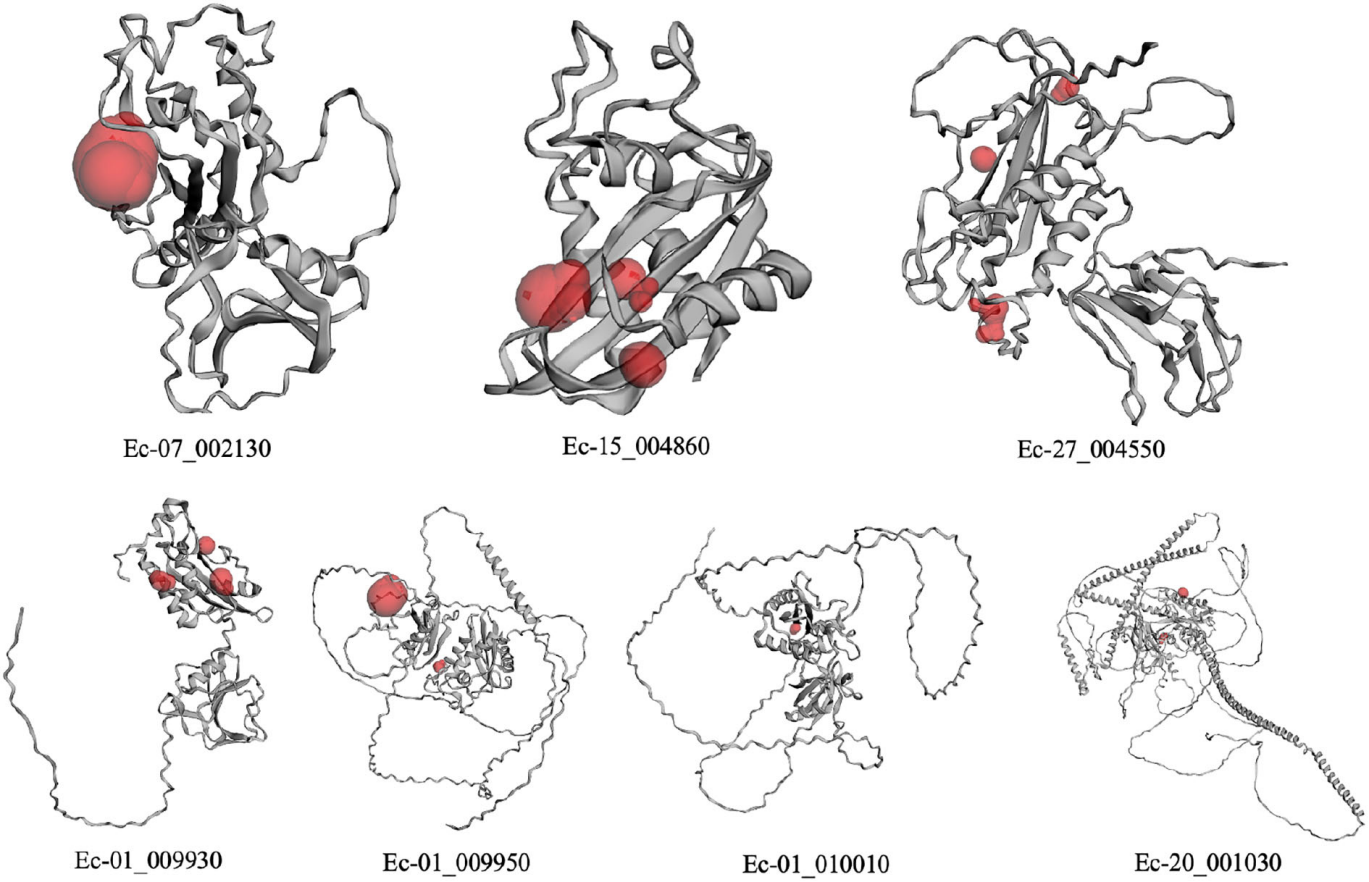
Predicted 3D structure of the EsPR-1 proteins from online alphafold protein database. Pockets were predicted using the CASTp 3.0 server and indicated in red.

The predicted 3D structures of the EsPR-1s were downloaded from the Alphafold Protein Structure Database. The structures show conserved four α-helices and four β-strands at the CAP domain, while extended structures exist in some sequences (**Figure 6**). For example, many random coil regions are predicted in Ec-01_009930, Ec-01_009950, Ec-01_010010 and Ec-20_001030. Notably, many extended α-helices are predicted in Ec-20_001030. Using the CASTp 3.0 server, molecular pockets in the 3D structures were identified. Each protein has one to three large pockets, which could indicate interaction sites for binding partners. According to the BetaCavityWeb server, the predicted void and channel number ranged from 7 to 31, 1 to 14, respectively. The least voids and channels (seven and one) were found in the shortest protein Ec-15_004860, whereas the most voids and channels number (31 and 19) were found in the longest protein Ec-20_001030 (**Supplementary Figure S2**). These structural variations may be connected with the diverse roles of the PR-1 proteins in *Ectocarpus*. Besides, modification conformation of proteins can occur via protein phosphorylation on serine, threonine, or tyrosine residues (Cutillas, 2017). We predicted the phosphorylation sites (PPS) using the NetPhos server. The PPS number ranged between 27 and 160 in EsPR-1s.

### Gene ontology and KEGG annotation

To explore the functional aspects of the genes, GO term annotation was executed for the 141 PR-1 proteins using the PANNZER online server. There are 51, 19 and 11 proteins that were assigned cellular component, biological process, and molecular function terms, respectively (**Supplementary Figure S3**). In terms of cellular components, most proteins show extracellular localization, which is consistent with subcellular location prediction. In terms of biological processes, proteins were assigned to cilium assembly, carbohydrate metabolism, cell adhesion and proteolysis. In terms of molecular function, some genes were assigned peptidase activity. Based on the GO annotation data, brown algal PR-1 proteins may function in various metabolic pathways. According to the KEGG annotation, 127 of the 141 PR-1 genes were assigned K numbers; for example, 57 genes were assigned the number K13449 (PR1), which corresponds to the MAPK signaling pathway (map04016), plant hormone signal transduction (map04075), and plant-pathogen interaction (map04626). 25 genes were assigned K24835 (GLIPR2), which corresponds to CAP domain-containing proteins. Some genes were linked to other categories, such as citrate cycle, lipoic acid metabolism, glycosidases, cell cycle, lectins and transporters, suggesting that these genes may participate in diverse functional pathways.

### Expression of PR-1 genes in brown algae

**Figure 7:**
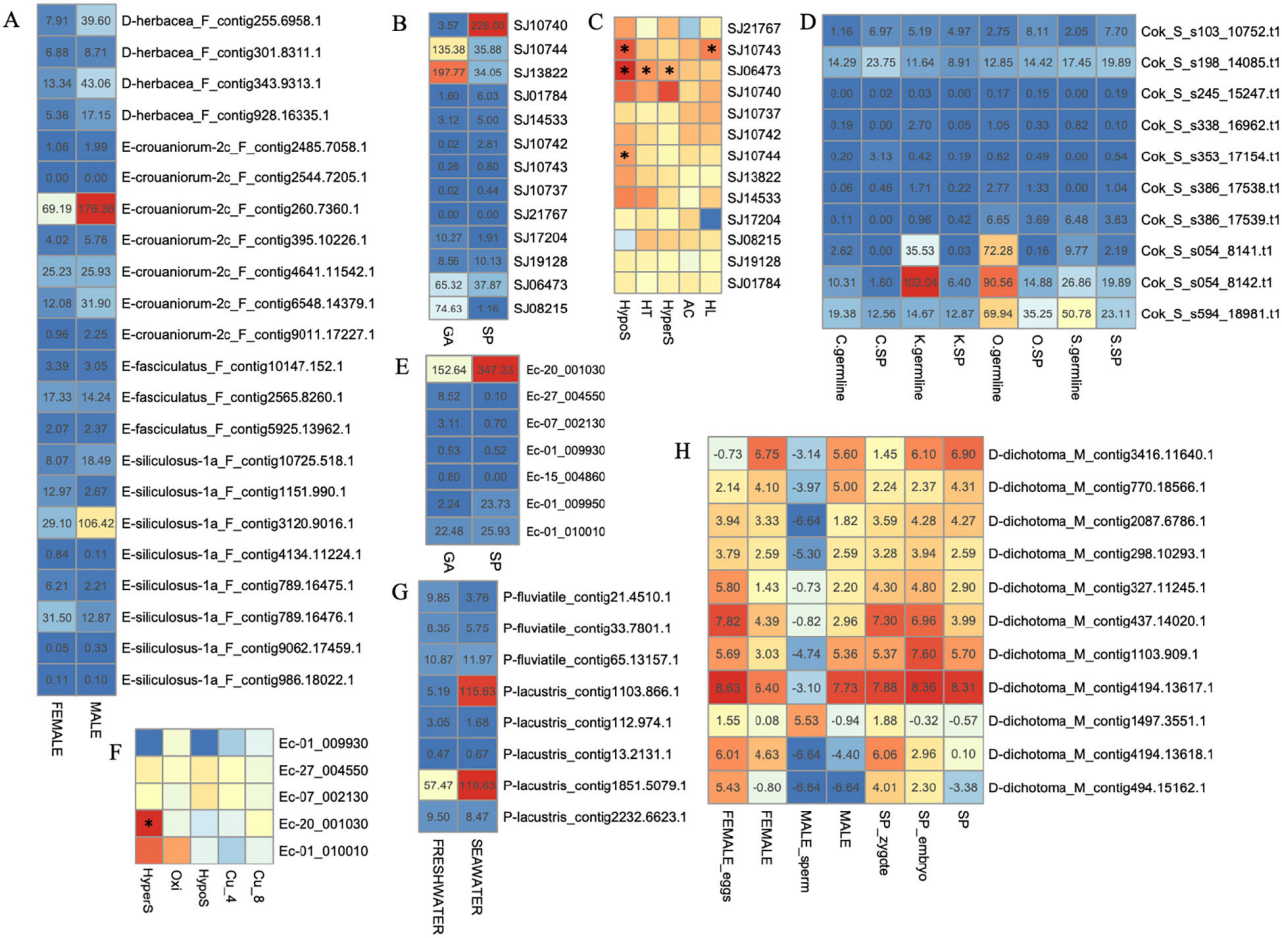
Expression profiles of PR-1 genes in brown algae. **(A–G)** TPM (transcripts per million) values of PR-1 genes in four brown algae. **(C, F)** Log2-transformed fold changes of the expression levels compared to the control. **(H)** Log2-transformed TPM in different developmental stage. Black stars indicate the significantly differently expressed genes, compared to the control (fold change >2, p <0.05, t-test).

To further understand the potential roles of PR-1 proteins in brown algae, we analyzed the expression profiles of the PR-1 genes in representative brown algae (**Figure 7**). For *Ectocarpus* sp., with five stress conditions analyzed, only one gene (Ec-20_001030) was upregulated significantly under hypersaline stress (fold change >2 and p-value<0.05), suggesting this gene may be involved in salt stress, while other genes were not sensitive to these abiotic stresses. For *S. japonica*, three genes were reported to have a significant response; among these, SJ06473 was upregulated under hyposaline, hypersaline, and high-temperature stresses, compared to the control, suggesting its roles in responses to the environment. SJ10743 was upregulated by hyposaline and high light stresses, and SJ10744 was upregulated by hyposaline stress. Notably, all the responsive genes were upregulated, and not downregulated under stress conditions, suggesting they should play positive roles in abiotic stress. However, abiotic stressors can lead to global changes in gene expression, so a gene being responsive to stressors may not indicate a direct role of the gene in the stress response (Teng et al., 2024). More targeted genetic methods are needed to elucidate their roles in the stresses. Most brown algae live in seawater, whereas *Pleurocladia lacustris* and *Porterinema fluviatile* not only occur as seawater species but are also two of the very rare freshwater representatives of the brown algae, giving it significant ecological and taxonomic importance. When comparing the species living in freshwater and seawater, two PR-1 genes of *P. lacustris* (contig1103.866.1, contig1851.5079.1) are highly expressed in seawater strain, suggestive of their potential roles in seawater environment.

We further explored the expression patterns at different developmental stages. In *Ectocarpus* sp., two genes (Ec-20_001030 and Ec-01_009950) were highly expressed in sporophytes (SP), and one gene (Ec-27_004550) was highly expressed in gametophytes (GA). Notably, Ec-20_001030, which belongs to the distinct clade 5, showed the most abundant expression in both SP and GA, and SP showed more than two-fold changes compared to GA, indicating its essential roles during SP development. For *S. japonica*, only one gene (SJ10740) was obviously SP-biased, with its expression level 75-fold higher than that in GA. By contrast, four genes (SJ10744, SJ13822, SJ06473 and SJ08215) were GA-biased, especially for SJ08215, with expression level 74-fold higher in GA compared to SP. Three GA-biased PR-1 genes (Cok_S_s054_8141.t1, Cok_S_s054_8142.t1, Cok_S_s594_18981.t1) were also found in all the four strains of *Cladosiphon okamuranus*, suggesting their key roles in GA development. Moreover, expression divergence also occurred between male and female GA. There are three, two and two male GA-biased genes in *D. hebacea, E. crouaniorum and E. siliculosus* respectively, while two female GA-biased genes were found in *E. siliculosus*. For *D. dichotoma*, expression changes were observed in more diverse life stages. Some genes, such as contig494.15162.1, contig437.14020.1, and contig4194.13618.1, showed higher expression levels in eggs than female GA, but showed a decreased trend during zygote development. On the contrary, contig3416.11640.1 showed no expression in eggs or sperms (TPM<1), while highly expressed in female and male GA, and showed an increasing expression from zygotes to mature SP. This spatial and temporal variability suggested potential roles of PR-1s in morphogenesis and growth of *D. dichotoma*. Notably, almost all PR-1 genes showed no expression in male sperms, except one gene from clade 5 contig1497.3551.1, which has the highest expression level in sperms, while no or slightly expressed in other stages, indicating its key roles in sperm development. Collectively, PR-1 genes showed divergent expression in different conditions and species of brown algae, suggesting their multiple roles in brown algae development.

## Discussion

PR proteins are a large group of proteins induced by pathogen attack, abiotic stress, or systemic acquired resistance (Van Loon et al., 2006). They appear to be an important component of the plant disease resistance mechanism, although the molecular functions are poorly defined. With the sequencing of plant genomes, the PR-1 gene family has been identified in many plant species, such as 12 in durum wheat (Zribi et al., 2023), 24 in soybean (Almeida-Silva and Venancio, 2022), 13 in tomato (Akbudak et al., 2020), 31 in pear (Wufuerjiang et al., 2025), 15 in banana (Anuradha et al., 2022), 11 in black pepper (Kattupalli et al., 2021), 17 in poplar (Wang et al., 2023), 23 in rice (Liu and Xue, 2006), 22 in Arabidopsis, 17 in tea (Zhang et al., 2022) and 20 in qingke barley (Yin et al., 2023). However, the PR-1 family has not been identified and analyzed in brown algae, which originated from secondary endosymbiosis events and are distant from plants. Taking advantage of brown algal genome project, we identified PR-1 proteins throughout the many brown algal species with high genome quality. Overall, the number of PR-1s in brown algae is less than plants. The surveyed plants often have PR-1s more than ten, up to 31 in pear. While the number of PR-1s in brown algae was found to range from 1 to 20, with the average of seven in each species. The number is much less in red algae, in which we found only zero to three PR-1s. PR1 proteins have been identified and characterized in many plants, and no correlation between the plant genome size and the number of identified PR-1 members has been found. Brown algae *Fucus serratus* and *Heterosigma akashiwo* have the large genome sizes of more than 1 billion base pairs (bp), and they were identified to have six and one PR-1 proteins, respectively. *Sargassum fusiforme* has the largest number of PR-1 proteins of 20, but its genome size is merely 394 million bp. Five species are predicted to code for more than 20 thousand proteins, but their identified PR-1 numbers are between three and 11, which suggests that the PR-1 numbers are not correlated with genome and proteome size (**Supplementary Table S3**). Comprehensive analysis of the phylogeny, gene structure, cis-elements, and expression patterns of PR-1s in brown algae were performed, which could provide scientific data for future functional research.

Studies found that PR-1 genes of plants carry one or no introns. For example, most PR1s of poplar, durum wheat, banana, apple, and qingke have one exon and no intron. However, for the two brown algae *Ectocarpus* sp. and *S. japonica*, the exon numbers of the PR-1 genes were found between two and 22, with an average number of seven. Generally, more introns were also found in other gene families of brown algae, such as MYB genes and ROCO genes (Zeng et al., 2022; Teng et al., 2024). For the brown algal genomes we surveyed, the genes in the genomes are intron-rich. The mean exon number per gene ranges from three to eleven, with an average number of 6.5 exons per gene (Denoeud et al., 2024). Compared with brown algae, land plants generally have lower average numbers of introns per gene in the genome; such as 5.43 in *Arabidopsis thaliana*, 4.39 in *Oryza sativa*, 3.89 in *Hordeum vulgare*, 4.35 in *Zea mays*, 5.34 in *Physcomitrella patens* and 5.69 in *Marchantia polymorpha*. Despite this, the *PR-1* genes of land plants rarely have introns. Rice and maize PR-1 type genes have no introns in their coding regions. The selective pressure theory may explain the intron loss of these genes in land plants. Intron density tended to be declined in some genes that need to produce proteins rapidly in response to external stimuli (Jeffares et al., 2006).

Analysis of biochemical properties revealed that 123 of the 141 brown algal PR-1s were classified as acidic, and only 18 as basic. Four species contain no basic PR-1s. Notably, this is in contrast with the results from many land plants. In tomato, 9 of 13 PR-1s were found to be basic. In tea, 10 of its 17 CsPR-1s were identified as basic. 13 of the 20 qingke PR-1s were basic. 12 of the 17 PtPR-1s in poplar were basic. Nine of the 11 PR-1s in *Piper nigrum* were basic. For PR-1 proteins, there is a link between their pI and anti-pathogenic properties (Yin et al., 2023). It was found that most PR-1s harboring anti-microbial properties tend to be basic. Basic PR-1c and PR-1g in tomato show stronger inhibitory effects against oomycetes, in comparison with acidic PR-1a (Niderman et al., 1995). Basic CsPR-1s were rapidly expressed under pathogen challenge (Zhang et al., 2022). Overexpression of the pepper basic PR-1 gene in tobacco has been shown to increase resistance to pathogens (Sarowar et al., 2005). Among the 22 PR-1 genes in *Arabidopsis*, only one PR-1 gene (At2g14610), which encodes a basic protein, is reported to be pathogen-responsive (Van Loon et al., 2006). In our present study, for the two brown algae, *S. japonica* and *Ectocarpus* sp., only one basic PR-1 was found in each species (SJ06473 and Ec-27_004550). Interestingly, among all the SjPR-1s, SJ06473 was the most responsive to environmental stress, it was upregulated under hyposaline, hypersaline, and high temperature stresses, suggesting its pivotal roles in environmental stress responses. *Pleurocladia lacustris* is an extremely rare, genuinely freshwater representative of the phylum Phaeophyta. When growing in seawater, the only one basic PR-1 (contig1103.866.1) showed extremely higher expression (more than 20-fold changes) compared with freshwater species, indicating its key roles in high salinity condition. Moreover, all the three basic PR-1 genes (SJ06473, Ec-27_004550, Cok_S_s594_18981.t1) were highly expressed in gametophytes, compared with sporophytes, suggesting the potential roles of basic PR-1 genes in the haploid life stage. Furthermore, when comparing the gene expression levels between male and female gametophytes, the only one basic PR-1 in each species *E. crouaniorum* (contig260.7360), *E. siliculosus* (contig10725.518.1) and *Desmarestia herbacea* (contig343.9313.1) show higher expression levels in male GA, further supporting the important roles of basic PR-1. Also, the only one basic PR-1 in *Dictyota dichotoma* (contig3416.11640.1) exhibits different expression during development, that is, no expression in eggs or sperms, while highly expressed in female and male GA, and increasingly expressed from zygotes to mature SP. In summary, the basic members of brown algal PR-1 family displayed obvious responses in different environmental and developmental conditions, and should be the focus of future research.

Our phylogenetic analysis revealed that PR-1 proteins in the brown algae (except clade 5) form a monophyletic group within the brown algal phylum and show homology and structural motifs in common with proteins from plants, fungi, and animals, making the PR-1 family a highly conserved group of proteins. Their widespread occurrence indicates that these proteins share a common evolutionary origin. However, clade 5 is distinct from the other four clades, based on phylogeny, sequence characteristics, and expression levels, suggesting its special evolutionary origin and function. In particular, the Ec-20_001030_and contig1497.3551.1 from clade 5 showed distinct responses under different conditions. Ec-20_001030 was significantly upregulated by hypersaline stress, and the gene in SP showed more than two-fold changes compared to GA. The contig1497.3551.1 of *D. dichotoma* showed the highest expression level in sperms, while no or slightly expressed in other stages. Although multiple pieces of evidence have indicated that PR-1s are defense proteins in plant-pathogen interactions, the biological functions of PR-1 proteins remain obscure. As stated above, only one PR-1 gene in *Arabidopsis* relates to pathogen resistance and the others may contribute to other functions. Some studies found that the PR-1 genes also respond to abiotic stresses such as light, cold, salt, and drought (Zeier et al., 2004). Moreover, as a member of CAP family, many glioma pathogenesis-related protein 1 (GLIPR1) family members from animals are homologous to the brown algal PR-1 proteins. GLIPR1 is considered to play important roles in the immune defense system, based on its similarity to plant PR-1s, high expression in macrophages and other immune cells, and was further found to participate in both tumor growth and suppression (Wang et al., 2022). The phylogeny and similarity of animal GLIPR1, plant and brown algal PR-1s suggested a common ancestor and possible functional link in immunity. However, diverse domain combinations identified in different lineages suggest a functional differentiation of PR-1 genes during evolution. Emergence of the brown algal lineage is associated with enhanced protein domain rearrangement (Denoeud et al., 2024). The integration WSC domain and blect domains with the CAP domain, which was not found in other phyla, suggests additional functions of brown algal PR-1 proteins, which should be the subject of future research. In the interaction networks of PR-1s in *Ectocarpus*, many carbohydrate metabolic enzymes were found to be involved, such as malic oxidoreductase, FAD dependent oxidoreductase and phosphoenolpyruvate carboxylase (**Supplementary Figure S4**). Furthermore, the WSC, blect and CAP domains are all involved in carbohydrate binding, further indicating their involvement in carbohydrate metabolic pathways. On the other hand, the expression pattern of *PR-1* genes in brown algae would greatly facilitate their functional annotation. The information on genes showing responses under different conditions should provide clues for function assignment. Clearly, *in vivo* experiments and *in vitro* studies are necessary to establish the activities and biological roles of brown algal *PR-1* genes.

## Conclusions

PR-1 family plays important roles in growth regulation, development, and plant response to biotic and abiotic stresses. In the present study, comprehensive analysis of PR-1 proteins in brown algae were performed. A total of 141 PR-1s were identified in the 19 brown algal genomes. Phylogenetic analysis showed that PR-1s in brown algae clustered into five clades, and showed no strong relationship with other lineages, suggesting an ancient origin. Their promoter regions were enriched in stress-response elements, hormone-response elements, and growth and development elements. Most PR-1s are acidic, while only 18 PR-1s are basic. Notably, expression analysis shows that the basic PR-1s maybe play important roles in abiotic stress conditions and of different life stages development. Our results provide valuable data for further research on the function of brown algal PR-1 family genes.

## Data Availability

The original contributions presented in the study are included in the article/**Supplementary Material**. Further inquiries can be directed to the corresponding author.
