# Supplementary material for: Genome-wide identification and analysis of expression of pathogenesis-related protein 1 (PR-1) gene family in brown algae
Source: Front Plant Sci. 2026 Jan 23;16:1754480. doi: 10.3389/fpls.2025.1754480 (PMC12879055; doi:10.3389/fpls.2025.1754480)
Supplement: Supplementary file 1 [file Table1.docx]

Supple table S1. The sequence information of the brown algal PR-1 genes.

| Sequence ID | MW（kDa） | pI | TH | Length(aa) | SP | SL | PPS |
| --- | --- | --- | --- | --- | --- | --- | --- |
| Chordaria linearis | | | | | | |  |
| contig67.13983.1 | 54.2 | 4.36 | 0 | 502 | NO | **Nucleus** | 62 |
| contig67.13984.1 | 53.0 | 4.89 | 0 | 487 | NO | **Extracell** | 45 |
| contig85.16153.1 | 16.6 | 5.19 | 0 | 150 | NO | **Extracell** | 9 |
| contig3.7733.1 | 37.0 | 8.40 | 0 | 350 | NO | **Extracell** | 38 |
| contig72.14894.1 | 118.4 | 4.83 | 0 | 1071 | NO | **Cytoplasm. Nucleus** | 93 |
| Cladosiphon okamuranus | | | | | | |  |
| Cok_S_s054_8141.t1 | 65.9 | 4.19 | 0 | 665 | YES | **Cytoplasm. Extracell** | 79 |
| Cok_S_s054_8142.t1 | 56.8 | 4.55 | 0 | 531 | YES | **Extracell** | 47 |
| Cok_S_s103_10752.t1 | 26.7 | 4.34 | 0 | 251 | NO | **Extracell** | 30 |
| Cok_S_s198_14085.t1 | 36.9 | 9.37 | 0 | 333 | NO | **Extracell** | 39 |
| Cok_S_s245_15247.t1 | 16.5 | 4.82 | 0 | 150 | NO | **Extracell** | 10 |
| Cok_S_s338_16962.t1 | 81.5 | 4.13 | 0 | 754 | YES | **Cytoplasm. Extracell** | 80 |
| Cok_S_s353_17154.t1 | 92.0 | 4.42 | 0 | 843 | NO | **Extracell** | 88 |
| Cok_S_s386_17538.t1 | 33.4 | 4.56 | 0 | 308 | YES | **Extracell** | 20 |
| Cok_S_s386_17539.t1 | 40.1 | 4.39 | 0 | 378 | YES | **Extracell** | 35 |
| Cok_S_s594_18981.t1 | 85.7 | 9.14 | 0 | 756 | NO | **Extracell** | 92 |
| Desmarestia herbacea | | | | | | | |
| contig343.9313.1 | 33.9 | 10.04 | 0 | 305 | NO | **Extracell** | 48 |
| contig928.16335.1 | 68.0 | 5.30 | 0 | 619 | YES | **Extracell. Nucleus** | 64 |
| contig255.6958.1 | 15.5 | 4.83 | 0 | 146 | NO | **Extracell** | 14 |
| contig301.8311.1 | 55.3 | 4.97 | 0 | 518 | YES | **Extracell** | 66 |
| Dictyota dichotoma | | | | | | | |
| contig327.11245.1 | 54.2 | 4.04 | 1 | 492 | YES | **Extracell** | 55 |
| contig1497.3551.1 | 135.7 | 4.98 | 0 | 1229 | NO | **Cytoplasm Nucleus** | 110 |
| contig4194.13617.1 | 20.5 | 4.05 | 0 | 189 | YES | **Extracell** | 15 |
| contig4194.13618.1 | 17.4 | 4.54 | 0 | 157 | NO | **Extracell** | 15 |
| contig770.18566.1 | 50.3 | 5.78 | 0 | 486 | NO | **Extracell** | 30 |
| contig494.15162.1 | 17.5 | 4.63 | 0 | 157 | NO | **Extracell** | 15 |
| contig2087.6786.1 | 25.3 | 4.01 | 0 | 238 | NO | **Extracell** | 32 |
| contig3416.11640.1 | 35.1 | 8.55 | 0 | 317 | NO | **Extracell** | 42 |
| contig1103.909.1 | 20.6 | 4.33 | 0 | 190 | YES | **Extracell** | 19 |
| contig437.14020.1 | 43.8 | 4.94 | 0 | 390 | YES | **Extracell** | 46 |
| contig298.10293.1 | 31.8 | 5.82 | 0 | 291 | NO | **Extracell** | 29 |
| Ectocarpus crouaniorum | | | | | | | |
| contig6548.14379.1 | 35.3 | 4.50 | 0 | 321 | YES | **Extracell** | 42 |
| contig9011.17227.1 | 32.6 | 4.32 | 0 | 291 | YES | **Extracell** | 36 |
| contig2544.7205.1 | 16.6 | 5.40 | 0 | 148 | NO | **Extracell** | 18 |
| contig395.10226.1 | 128.7 | 4.69 | 0 | 1169 | NO | **Cytoplasm. Nucleus** | 94 |
| contig260.7360.1 | 44.1 | 9.77 | 0 | 402 | NO | **Extracell** | 41 |
| contig2485.7058.1 | 49.4 | 4.10 | 0 | 459 | YES | **Cytoplasm** | 56 |
| contig4641.11542.1 | 15.2 | 4.21 | 0 | 149 | NO | **Extracell** | 12 |
| Ectocarpus fasciculatus | | | | | | | |
| contig10147.152.1 | 31.5 | 4.19 | 0 | 283 | YES | **Extracell** | 33 |
| contig5925.13962.1 | 47.3 | 4.10 | 0 | 435 | YES | **Cytoplasm** | 52 |
| contig2565.8260.1 | 49.2 | 4.27 | 0 | 469 | YES | **Extracell** | 68 |
| Ectocarpus siliculosus | | | | | | | |
| contig3120.9016.1 | 27.9 | 4.53 | 0 | 255 | YES | **Cell membrane. Extracell** | 38 |
| contig986.18022.1 | 57.0 | 4.79 | 0 | 536 | NO | **Extracell. Nucleus.** | 76 |
| contig789.16475.1 | 70.7 | 4.05 | 0 | 666 | NO | **Cytoplasm. Extracell.** | 82 |
| contig789.16476.1 | 84.4 | 4.15 | 0 | 788 | NO | **Cytoplasm. Extracell** | 104 |
| contig9062.17459.1 | 47.0 | 4.26 | 0 | 442 | NO | **Nucleus** | 62 |
| contig1151.990.1 | 130.0 | 4.65 | 0 | 1183 | NO | **Cytoplasm. Nucleus** | 103 |
| contig10725.518.1 | 16.6 | 7.25 | 0 | 152 | NO | **Extracell** | 24 |
| contig4134.11224.1 | 16.6 | 5.57 | 0 | 148 | NO | **Extracell** | 16 |
| Fucus serratus | | | | | | | |
| contig292.9448.1 | 39.9 | 4.15 | 0 | 375 | NO | **Extracell** | 56 |
| contig1828.5108.1 | 52.2 | 4.44 | 1 | 482 | YES | **Extracell** | 42 |
| contig103.339.1 | 133.2 | 4.74 | 0 | 1194 | NO | **Cytoplasm. Nucleus** | 98 |
| contig183.5131.1 | 23.1 | 4.29 | 0 | 218 | YES | **Extracell** | 18 |
| contig361.11525.1 | 31.3 | 4.11 | 0 | 283 | NO | **Extracell** | 31 |
| contig31.10032.1 | 42.1 | 8.88 | 0 | 386 | NO | **Extracell** | 51 |
| Heterosigma akashiwo | | | | | | | |
| Contig178.2.1 | 22.0 | 5.69 | 0 | 197 | NO | **Extracell** | 19 |
| Sargassum fusiforme | | | | | | | |
| g1486.t1 | 39.9 | 9.43 | 0 | 352 | NO | **Extracell** | 48 |
| g1489.t1 | 30.5 | 4.57 | 0 | 278 | NO | **Extracell** | 18 |
| g7349.t1 | 36.1 | 4.12 | 0 | 337 | NO | **Extracell** | 50 |
| g7350.t1 | 41.8 | 4.16 | 1 | 389 | NO | **Extracell** | 55 |
| g8435.t1 | 43.1 | 4.36 | 0 | 404 | YES | **Extracell** | 62 |
| g9439.t1 | 14.9 | 3.92 | 0 | 136 | NO | **Extracell** | 8 |
| g9441.t1 | 25.7 | 4.29 | 1 | 233 | YES | **Extracell** | 24 |
| g9443.t1 | 16.5 | 3.99 | 0 | 149 | NO | **Extracell** | 9 |
| g11912.t1 | 98.9 | 4.68 | 0 | 889 | NO | **Cytoplasm. Extracell** | 92 |
| g12440.t1 | 31.4 | 4.53 | 0 | 285 | NO | **Extracell** | 25 |
| g12441.t1 | 30.5 | 4.58 | 0 | 279 | NO | **Extracell** | 17 |
| g12442.t1 | 31.3 | 4.73 | 0 | 283 | NO | **Extracell** | 19 |
| g13156.t1 | 16.2 | 6.18 | 0 | 149 | NO | **Extracell** | 15 |
| g14022.t1 | 22.4 | 4.47 | 0 | 207 | NO | **Extracell** | 16 |
| g15356.t1 | 43.3 | 4.83 | 0 | 395 | NO | **Extracell** | 36 |
| g15357.t1 | 44.7 | 4.83 | 0 | 412 | NO | Extracell. | 64 |
| g15358.t1 | 44.8 | 4.62 | 0 | 414 | YES | Extracell. | 51 |
| g15896.t1 | 39.0 | 4.14 | 0 | 365 | YES | Extracell. | 58 |
| g17454.t1 | 24.0 | 3.94 | 0 | 228 | NO | Extracell. | 52 |
| g17456.t1 | 40.7 | 4.11 | 0 | 380 | YES | Extracell. | 79 |
| Pleurocladia lacustris | | | | | | | |
| contig1851.5079.1 | 27.3 | 4.79 | 0 | 257 | NO | Extracell. | 52 |
| contig2232.6623.1 | 56.8 | 4.32 | 0 | 527 | YES | Extracell. | 79 |
| contig112.974.1 | 64.2 | 4.35 | 0 | 601 | NO | Extracell. | 94 |
| contig1103.866.1 | 15.7 | 9.07 | 0 | 144 | NO | Extracell. | 18 |
| contig13.2131.1 | 17.4 | 5.80 | 0 | 154 | NO | Extracell. | 21 |
| Porterinema fluviatile | | | | | | | |
| contig33.7801.1 | 16.2 | 5.24 | 0 | 151 | NO | Extracell. | 29 |
| contig21.4510.1 | 117.6 | 5.04 | 0 | 1061 | NO | Cytoplasm. Nucleus. | 160 |
| contig65.13157.1 | 17.1 | 5.01 | 0 | 154 | NO | Extracell. | 17 |
| Pylaiella littoralis | | | | | | | |
| Contig46.179.8 | 130.5 | 4.78 | 0 | 1175 | NO | Cytoplasm. Nucleus. | 179 |
| Contig1321.3.1 | 41.2 | 4.49 | 0 | 401 | YES | Extracell. | 66 |
| Contig252.23.1 | 16.5 | 4.90 | 0 | 147 | NO | Extracell. | 25 |
| Contig296.9.5 | 71.8 | 4.50 | 0 | 716 | NO | Extracell. | 96 |
| Contig354.19.2 | 76.9 | 4.18 | 0 | 735 | YES | Extracell. Nucleus. | 179 |
| Contig63.66.2 | 48.7 | 4.68 | 0 | 471 | NO | Extracell. Nucleus. | 113 |
| Saccharina latissima | | | | | | | |
| contig139.2871.1 | 15.3 | 4.34 | 0 | 142 | NO | Extracell. | 40 |
| contig41.11283.1 | 38.7 | 9.32 | 0 | 346 | NO | Extracell. | 86 |
| contig1388.2863.1 | 16.5 | 4.69 | 0 | 153 | NO | Extracell. | 23 |
| contig9.16781.1 | 118.4 | 4.62 | 0 | 1084 | NO | Cytoplasm. Nucleus. | 154 |
| contig1417.3047.1 | 15.3 | 4.66 | 0 | 139 | NO | Extracell. | 24 |
| Schizocladia ischiensis | | | | | | | |
| contig28.8504.1 | 27.4 | 5.01 | 0 | 246 | NO | Extracell. | 33 |
| contig40.12277.1 | 26.2 | 7.09 | 1 | 237 | NO | Extracell. | 44 |
| contig77.19029.1 | 112.9 | 4.78 | 0 | 1053 | NO | Cytoplasm. | 118 |
| Scytosiphon promiscuus | | | | | | | |
| Contig872.1.1 | 29.5 | 4.20 | 0 | 278 | NO | Extracell. | 65 |
| Contig18375.4.1 | 24.6 | 10.05 | 0 | 222 | NO | Extracell. | 15 |
| Contig129.27.2 | 74.1 | 3.92 | 0 | 703 | NO | Extracell. | 173 |
| Contig2472.2.1 | 16.2 | 5.61 | 0 | 146 | NO | Extracell. | 19 |
| Contig800.7.1 | 54.7 | 4.23 | 0 | 506 | NO | Extracell. | 75 |
| Contig2068.11.3 | 113.1 | 4.80 | 0 | 1022 | NO | Cytoplasm. Nucleus. | 160 |
| Contig67162.1.1 | 19.8 | 5.29 | 0 | 185 | YES | Extracell. | 15 |
| Contig921.13.1 | 34.6 | 4.75 | 0 | 321 | NO | Extracell. | 89 |
| Contig77.26.1 | 67.2 | 4.06 | 0 | 635 | YES | Extracell. Nucleus. | 177 |
| Contig1181.2.2 | 57.1 | 4.35 | 0 | 532 | YES | Extracell. | 80 |
| Contig1673.9.1 | 41.2 | 10.01 | 0 | 369 | NO | Extracell. | 79 |
| Saccharina japonica | | | | | | | |
| SJ01784 | 25.4 | 4.34 | 0 | 243 | NO | Extracell. | 44 |
| SJ06473 | 37.7 | 8.80 | 0 | 339 | NO | Extracell. | 70 |
| SJ08215 | 57.8 | 4.56 | 0 | 545 | YES | Extracell. | 98 |
| SJ10737 | 38.8 | 5.35 | 0 | 368 | NO | Cell membrane. Extracell. | 54 |
| SJ10740 | 28.5 | 4.44 | 0 | 264 | YES | Extracell. Nucleus. | 87 |
| SJ10744 | 18.0 | 5.66 | 0 | 164 | NO | Extracell. | 17 |
| SJ10742 | 33.8 | 6.22 | 0 | 311 | YES | Extracell. | 48 |
| SJ10743 | 21.2 | 6.21 | 0 | 198 | YES | Extracell. | 30 |
| SJ13822 | 25.2 | 4.10 | 0 | 234 | NO | Extracell. | 53 |
| SJ14533 | 43.6 | 4.25 | 0 | 411 | NO | Extracell. | 83 |
| SJ17204 | 61.1 | 4.70 | 1 | 578 | YES | Extracell. | 101 |
| SJ19128 | 57.5 | 4.15 | 0 | 541 | NO | Extracell. | 115 |
| SJ21767 | 20.2 | 5.13 | 0 | 187 | NO | Extracell. | 40 |
| Ectocarpus sp. | | | | | | | |
| Ec-01_009930 | 34.8 | 4.49 | 0 | 319 | YES | Extracell. | 69 |
| Ec-01_009950 | 61.2 | 4.67 | 0 | 579 | NO | Extracell. Nucleus. | 160 |
| Ec-01_010010 | 63.6 | 4.99 | 0 | 602 | NO | Cell membrane. Extracell. Nucleus. | 145 |
| Ec-07_002130 | 27.1 | 4.23 | 0 | 262 | NO | Extracell. | 35 |
| Ec-15_004860 | 16.8 | 5.40 | 0 | 150 | NO | Extracell. | 27 |
| Ec-20_001030 | 125.3 | 4.63 | 0 | 1138 | NO | Cytoplasm. Nucleus. | 141 |
| Ec-27_004550 | 39.2 | 9.62 | 0 | 355 | NO | Extracell. | 63 |
| Nemacystus decipiens | | | | | | | |
| g421.t1 | 71.6 | 7.22 | 0 | 683 | NO | Chloroplast. Cytoplasm. | 63 |
| g1407.t1 | 98.0 | 8.95 | 0 | 911 | NO | Extracell. | 150 |
| g3562.t1 | 105.8 | 4.15 | 0 | 976 | YES | Cytoplasm. Nucleus. | 184 |
| g7294.t1 | 89.8 | 4.29 | 1 | 824 | YES | Extracell. Nucleus. | 176 |
| g7295.t1 | 66.1 | 4.18 | 1 | 620 | YES | Extracell. | 137 |
| g7313.t1 | 48.4 | 4.26 | 0 | 454 | YES | Extracell. | 75 |
| g7315.t1 | 48.6 | 4.28 | 0 | 454 | YES | Extracell. | 69 |
| g7316.t1 | 106.1 | 4.31 | 0 | 985 | NO | Extracell. | 157 |
| g12090.t1 | 30.8 | 3.95 | 0 | 287 | YES | Extracell. | 47 |
| g12091.t1 | 46.5 | 4.20 | 0 | 437 | NO | Extracell. Nucleus. | 107 |
| g12096.t1 | 97.6 | 4.01 | 0 | 916 | NO | Extracell. | 192 |
| g12621.t1 | 215.2 | 4.43 | 3 | 1994 | YES | Cytoplasm.  Extracell. | 473 |
| g13234.t1 | 15.9 | 5.05 | 0 | 144 | NO | Extracell. | 15 |

SL, subcellular location; PPS, phosphorylation sites; SP, signal peptide; TH, transmembrane helixes; PI, theoretical isoelectric point; MW, molecular weight

**Suppl. Table S2.** The number of cis-elements identified in the PR-1 promoter regions in Saccharina japonica. The cis-element numbers from each function response to stress, hormone, growth and development, transcription factors binding were listed.

| Function | motif | | SJ10744 | | SJ10743 | | SJ10742 | | | SJ21767 | | | SJ10737 | | SJ10740 | | SJ13822 | | SJ08215 | SJ01784 | SJ17204 | | | SJ19128 | | SJ06473 | SJ14533 |  |  |  |
| --- | --- | --- | --- | --- | --- | --- | --- | --- | --- | --- | --- | --- | --- | --- | --- | --- | --- | --- | --- | --- | --- | --- | --- | --- | --- | --- | --- | --- | --- | --- |
| **stress response** |  | |  | |  | |  | | |  | | |  | |  | |  | |  |  |  | | |  | |  |  |  |  |  |
| anaerobic induction | ARE | | 1 | | 3 | | 1 | | | 1 | | |  | |  | | 1 | | 1 | 1 | 1 | | | 1 | | 1 | 2 |  |  |  |
| low-temperature responsive | LTR | | 4 | |  | | 2 | | | 3 | | | 2 | | 2 | | 1 | | 4 | 1 | 1 | | | 1 | |  | 2 |  |  |  |
| defense and stress responsive | TC-rich | |  | | 2 | | 1 | | | 1 | | | 1 | | 1 | |  | |  |  |  | | |  | |  |  |  |  |  |
| anoxic specific inducibility | GC-motif | |  | |  | | 1 | | |  | | | 3 | |  | |  | |  | 4 | 2 | | | 1 | | 2 | 1 |  |  |  |
| **hormone response** |  | |  | |  | |  | | |  | | |  | |  | |  | |  |  |  | | |  | |  |  |  |  |  |
| MeJA responsive | CGTCA-motif | | 2 | | 3 | | 5 | | | 6 | | | 2 | | 1 | | 5 | | 1 | 2 | 4 | | | 1 | | 5 | 6 |  |  |  |
|  | TGACG-motif | | 2 | | 3 | | 5 | | | 6 | | | 2 | | 1 | | 5 | | 1 | 2 | 4 | | | 1 | | 5 | 6 |  |  |  |
| auxin responsive | TGA-element | | 2 | | 2 | | 3 | | | 2 | | | 1 | | 3 | | 2 | | 2 | 1 | 2 | | | 1 | | 1 | 2 |  |  |  |
| abscisic acid responsive | ABRE | | 3 | | 4 | | 8 | | | 3 | | | 2 | | 2 | | 3 | | 6 | 5 | 4 | | | 2 | | 9 | 6 |  |  |  |
| salicylic acid responsive | TCA-element | | 1 | | 1 | |  | | |  | | | 1 | | 1 | |  | | 3 |  | 2 | | |  | |  | 1 |  |  |  |
| gibberellin responsive | P-box | |  | |  | |  | | |  | | |  | |  | |  | | 1 | 1 |  | | | 1 | |  |  |  |  |  |
|  | TATC-box | |  | |  | |  | | |  | | | 1 | |  | |  | |  | 1 |  | | |  | |  |  |  |  |  |
|  | GARE-motif | |  | |  | | |  | | | 1 | |  | |  | | 1 | |  | | | | | | | |  |  | 1 |  |
| **growth and development** |  | |  | |  | |  | | |  | | |  | |  | |  | |  |  |  | | |  | |  |  |  |  |  |
| meristem expression | CAT-box | | 2 | |  | | 1 | | | 1 | | |  | |  | |  | |  |  |  | | |  | |  | 3 |  |  |  |
| endosperm expression | GCN4-motif | | 1 | |  | | |  | | |  | |  | |  | |  | |  | | | | | | | |  |  |  |  |
| circadian control | circadian | |  | |  | |  | | |  | | |  | |  | |  | | 1 |  |  | | |  | | 1 |  |  |  |  |
| light responsive | AAAC-motif | | 1 | |  | |  | | |  | | |  | |  | |  | |  |  |  | | |  | |  |  |  |  |  |
|  | ACE | |  | |  | |  | | | 1 | | | 1 | | 1 | |  | |  | 1 | 1 | | |  | |  | 2 |  |  |  |
|  | AE-box | |  | |  | |  | | |  | | |  | |  | | 1 | | 2 |  | 1 | | |  | |  |  |  |  |  |
|  | ATCT-motif | |  | |  | | |  | | | 1 | |  | |  | |  | |  | | | | | | | |  |  |  |  |
|  | C-box | |  | |  | |  | | |  | | |  | |  | |  | | 1 |  |  | | |  | |  |  |  |  |  |
|  | CAG-motif | |  | |  | |  | | |  | | |  | |  | |  | |  |  |  | | |  | | 1 |  |  |  |  |
|  | G-box | | 3 | | 4 | | 7 | | | 7 | | | 3 | | 1 | | 3 | | 5 | 4 | 4 | | | 2 | | 6 | 6 |  |  |  |
|  | GATA-motif | |  | | 1 | | |  | | |  | |  | | 1 | | 1 | |  | | | | | | | |  | 2 |  |  |
|  | GT1-motif | |  | | 2 | | 1 | | | 3 | | | 5 | |  | |  | |  | 1 | 2 | | | 1 | |  |  |  |  |  |
|  | GTGGC-motif | |  | |  | | |  | | | 1 | |  | |  | |  | |  | | | | | | | |  |  |  |  |
|  | Gap-box | |  | |  | |  | | |  | | |  | |  | |  | |  |  | 1 | | |  | |  |  |  |  |  |
|  | I-box | |  | |  | |  | | | 1 | | |  | |  | |  | |  |  |  | | |  | | 2 | 1 |  |  |  |
|  | LAMP-element | | 1 | |  | | |  | | |  | |  | |  | |  | |  | | | | | | | |  |  |  |  |
|  | MRE | |  | | 1 | |  | | |  | | |  | |  | |  | |  |  |  | | |  | |  |  |  |  |  |
|  | Sp1 | | 1 | | 1 | | 2 | | | 2 | | |  | |  | |  | | 1 | 3 |  | | |  | |  | 2 |  |  |  |
|  | TCCC-motif | |  | |  | | | 1 | | |  | |  | | 1 | | 1 | |  | | | | | | | |  |  |  |  |
|  | TCT-motif | |  | | 2 | |  | | |  | | | 1 | |  | | 1 | | 1 | 1 |  | | |  | | 1 | 1 |  |  |  |
|  | chs-CMA2a | |  | |  | |  | | |  | | |  | |  | |  | |  |  | 2 | | |  | |  |  |  |  |  |
|  | sbp-CMA1c | |  | |  | | |  | | |  | |  | |  | | 1 | |  | | | | | | | |  |  |  |  |
| **transcription factor binding site** |  |  | |  | |  | | |  | | |  | |  | |  | |  |  |  | |  |  | |  |  |  |  |  |  |
| MYB binding |  | | 9 | | 20 | | 11 | | | 8 | | | 11 | | 11 | | 7 | | 12 | 6 | 2 | | | 1 | | 10 | 20 |  |  |  |

**Table S3**. The number of PR-1 proteins in each species, showing the acidic and basic protein number, the genome and proteome sizes, and the average exon number in the genomes.

|  | PR-1 | acidic | basic | genome | Pr number | Exon per gene |
| --- | --- | --- | --- | --- | --- | --- |
| Chordaria linearis | 5 | 4 | 1 | 214M | 17198 | 7.1 |
| Cladosiphon okamuranus | 10 | 8 | 2 | 140 | 13640 | 10.53 |
| Desmarestia herbacea | 4 | 3 | 1 | 484 | 16759 | 6.44 |
| Dictyota dichotoma | 11 | 10 | 1 | 851 | 20583 | 5.57 |
| Ectocarpus crouaniorum | 7 | 6 | 1 | 210 | 18132 | 5.38 |
| Ectocarpus fasciculatus | 3 | 3 | 0 | 165 | 18167 | 7.51 |
| Ectocarpus siliculosus | 8 | 7 | 1 | 186 | 18119 | 6.02 |
| Fucus serratus | 6 | 5 | 1 | 1.23G | 21263 | 6.07 |
| Heterosigma akashiwo | 1 | 1 | 0 | 1.52G | 15415 | 2.99 |
| Sargassum fusiforme | 20 | 19 | 1 | 394 | 19404 | 6.81 |
| Pleurocladia lacustris | 5 | 4 | 1 | 220 | 16268 | 5.82 |
| Porterinema fluviatile | 3 | 3 | 0 | 167 | 15519 | 7.13 |
| Pylaiella littoralis | 6 | 6 | 0 | 322 | 22501 | 5.87 |
| Saccharina latissima | 5 | 4 | 1 | 531 | 18169 | 5.92 |
| Schizocladia ischiensis | 3 | 2 | 1 | 194 | 21187 | 4.7 |
| Scytosiphon promiscuus | 11 | 9 | 2 | 256 | 30281 | 4.49 |
| Saccharina japonica | 13 | 12 | 1 | 537 | 18733 | 6.49 |
| Ectocarpus sp. | 7 | 6 | 1 | 214 | 16256 | 7.96 |
| Nemacystus decipiens | 13 | 11 | 2 | 154 | 15156 | 11.24 |


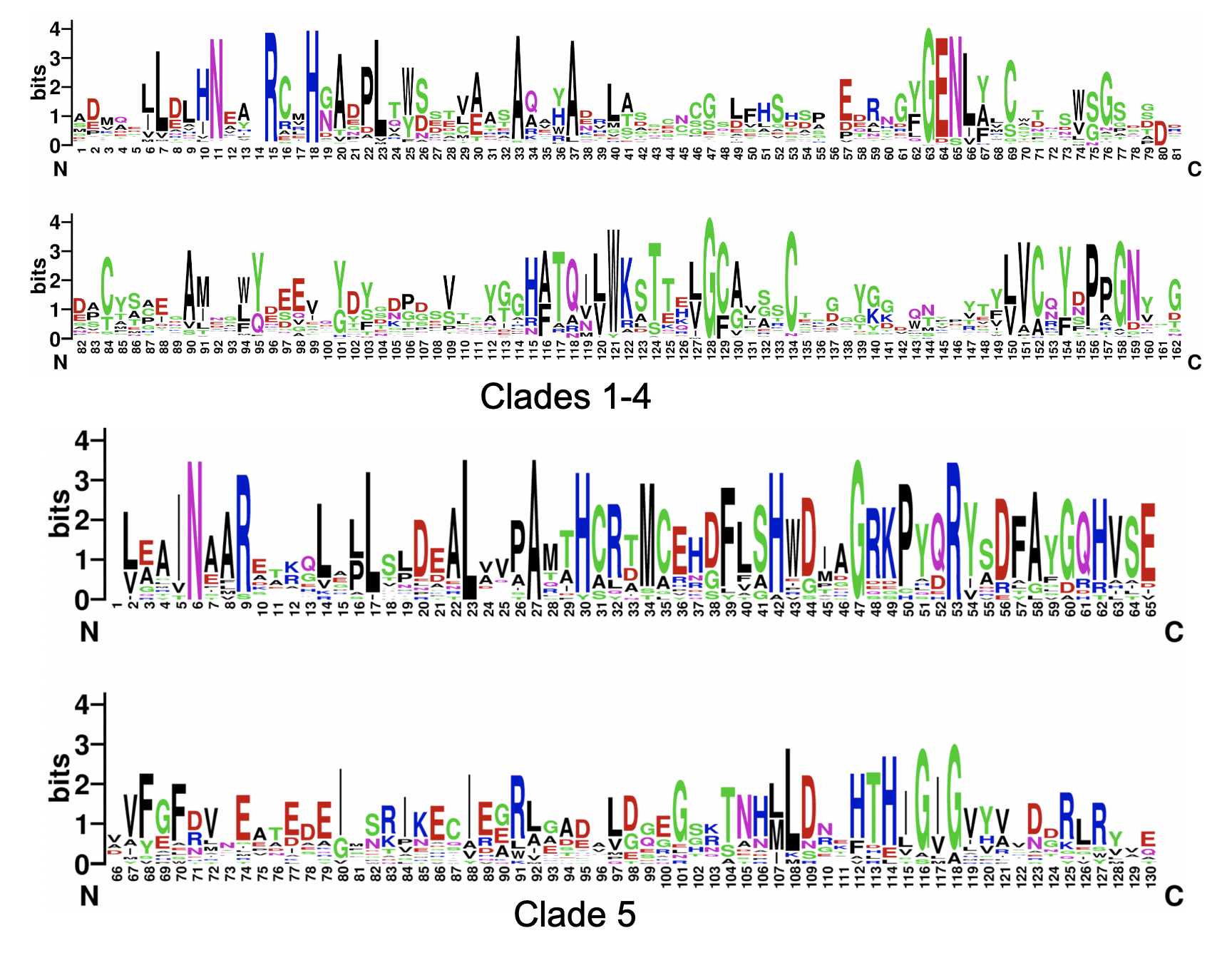


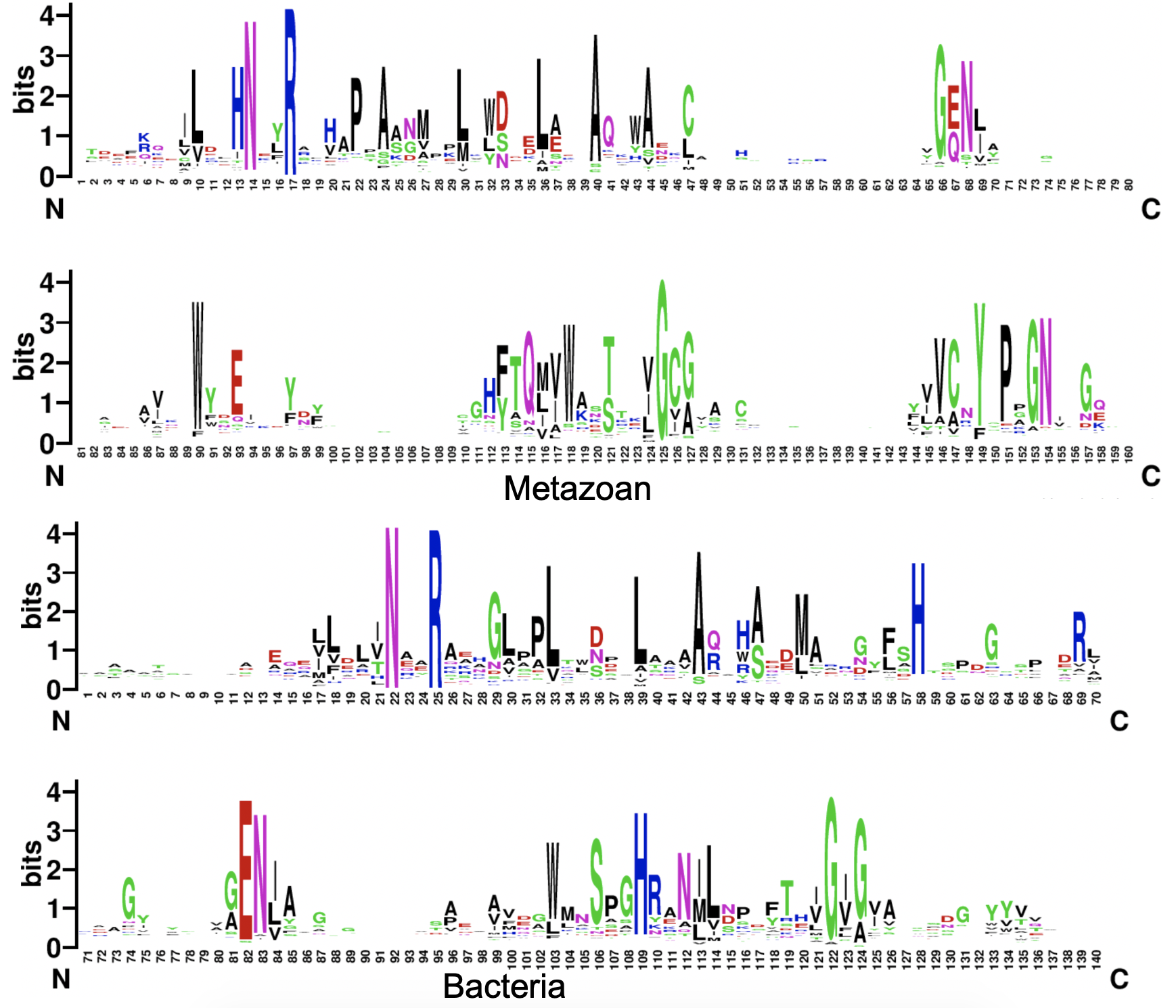


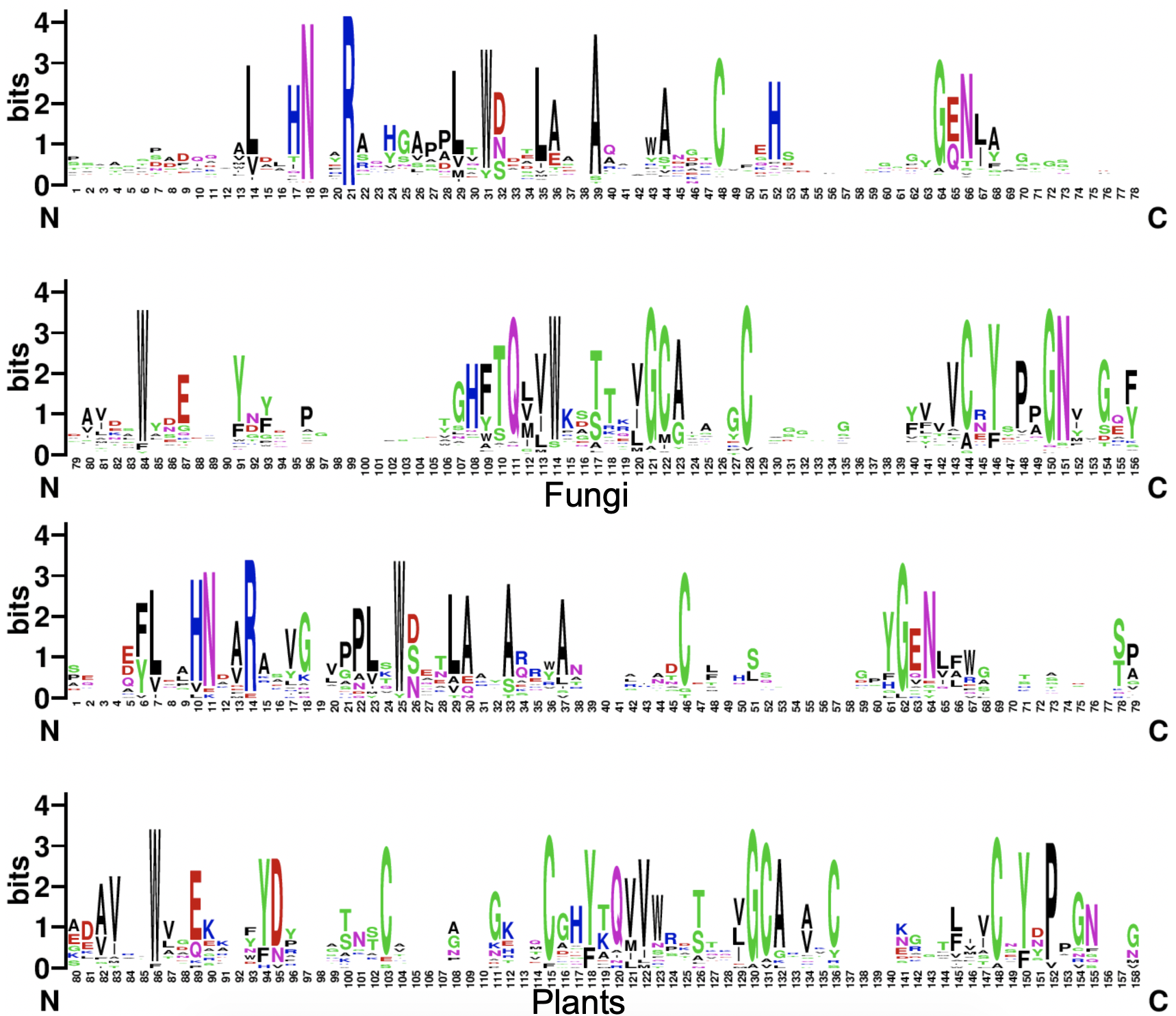


**Figure S1**. The sequence logo of the CAP domain in brown algae (clades 1-5) and different lineages.


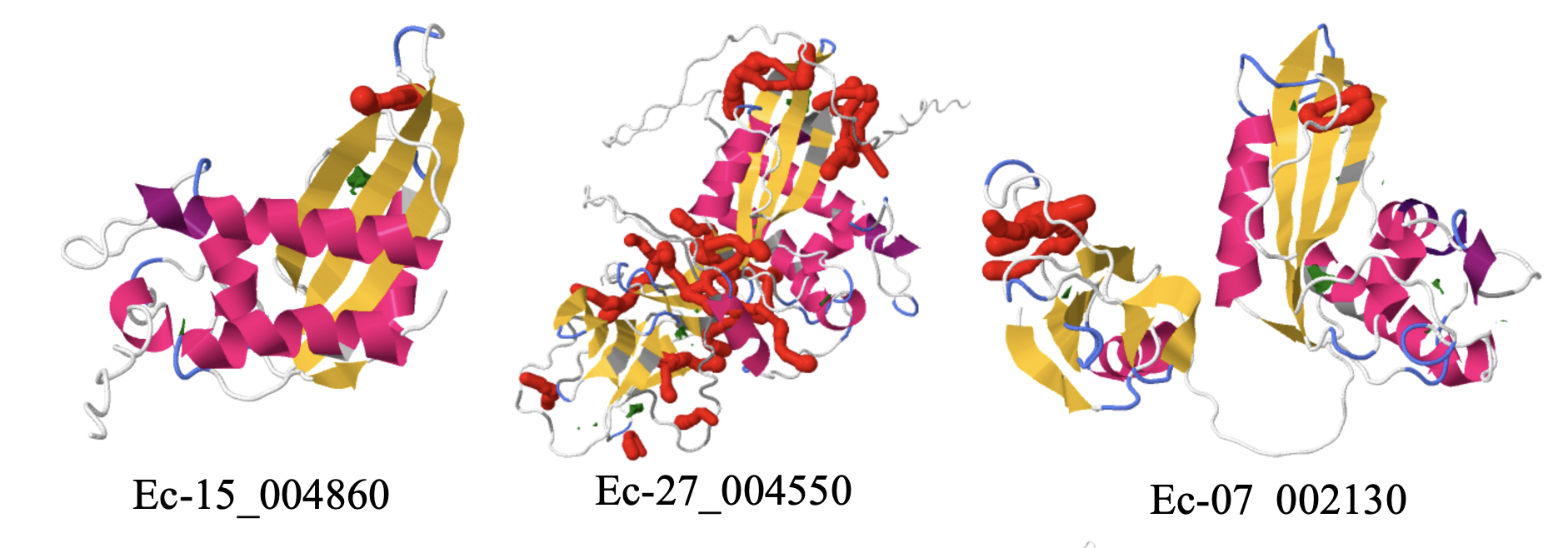


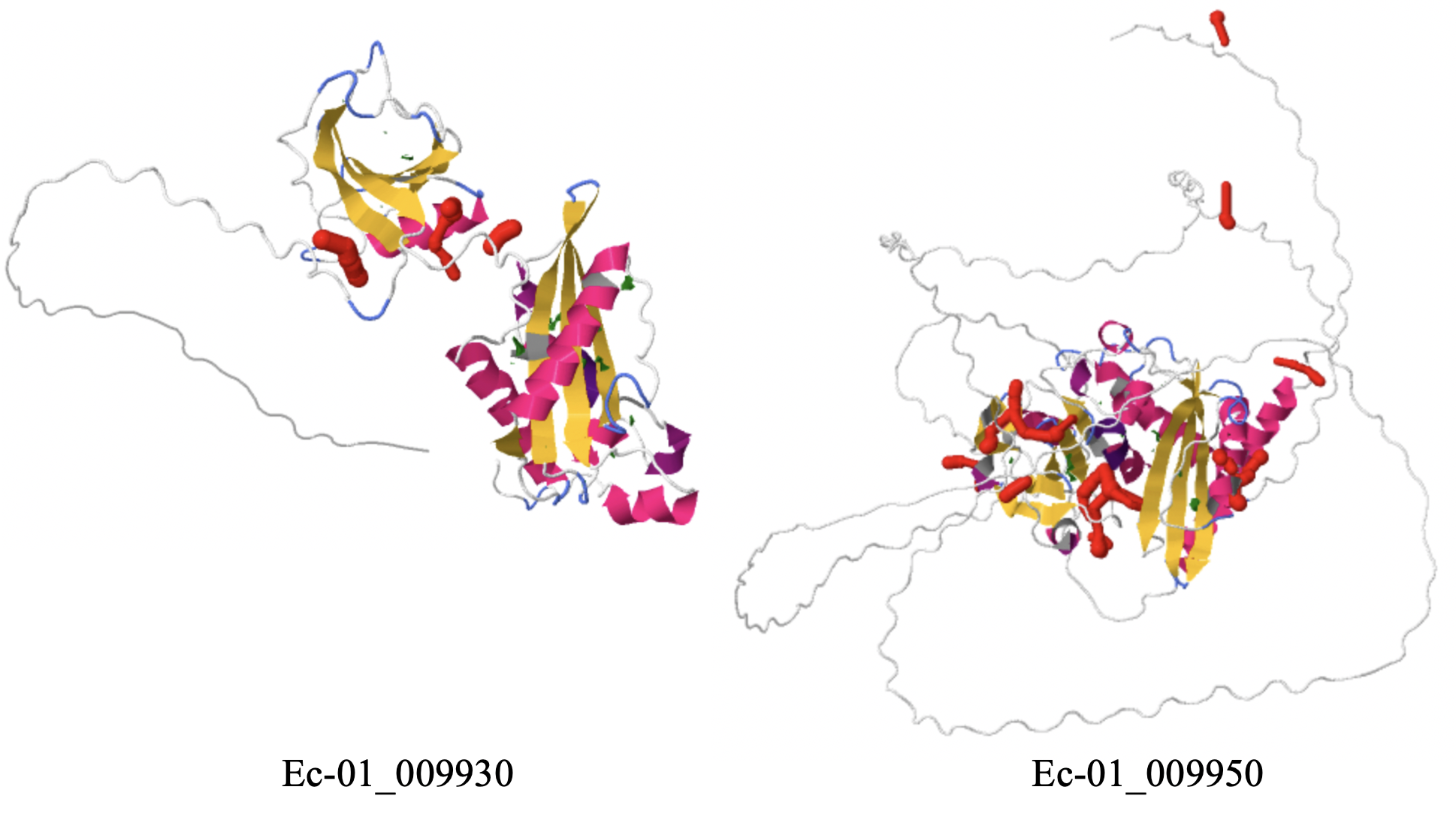


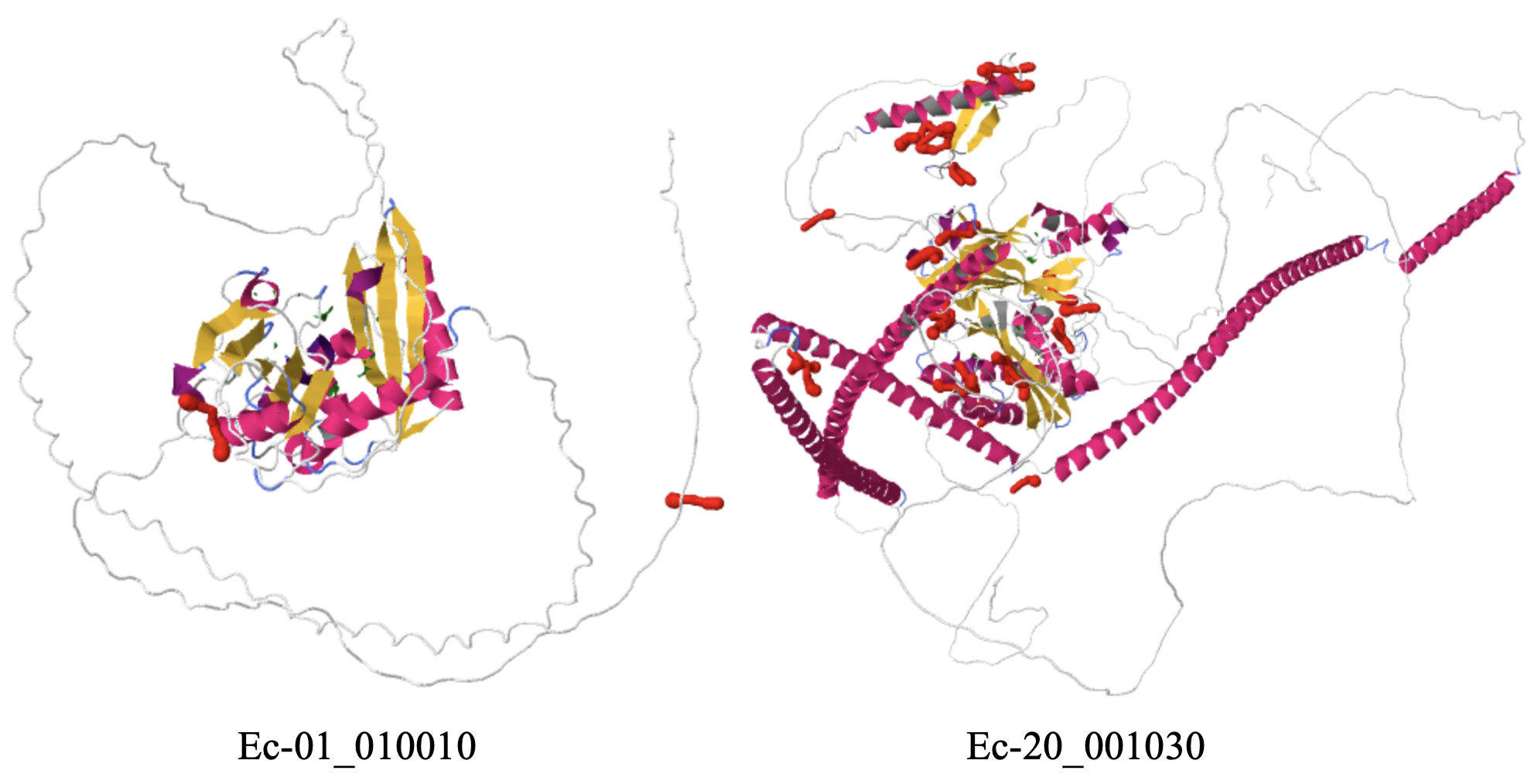


**Figure S2**. The predicted 3D structure of EsPR-1 proteins, showing the voids (green) and channels (red).


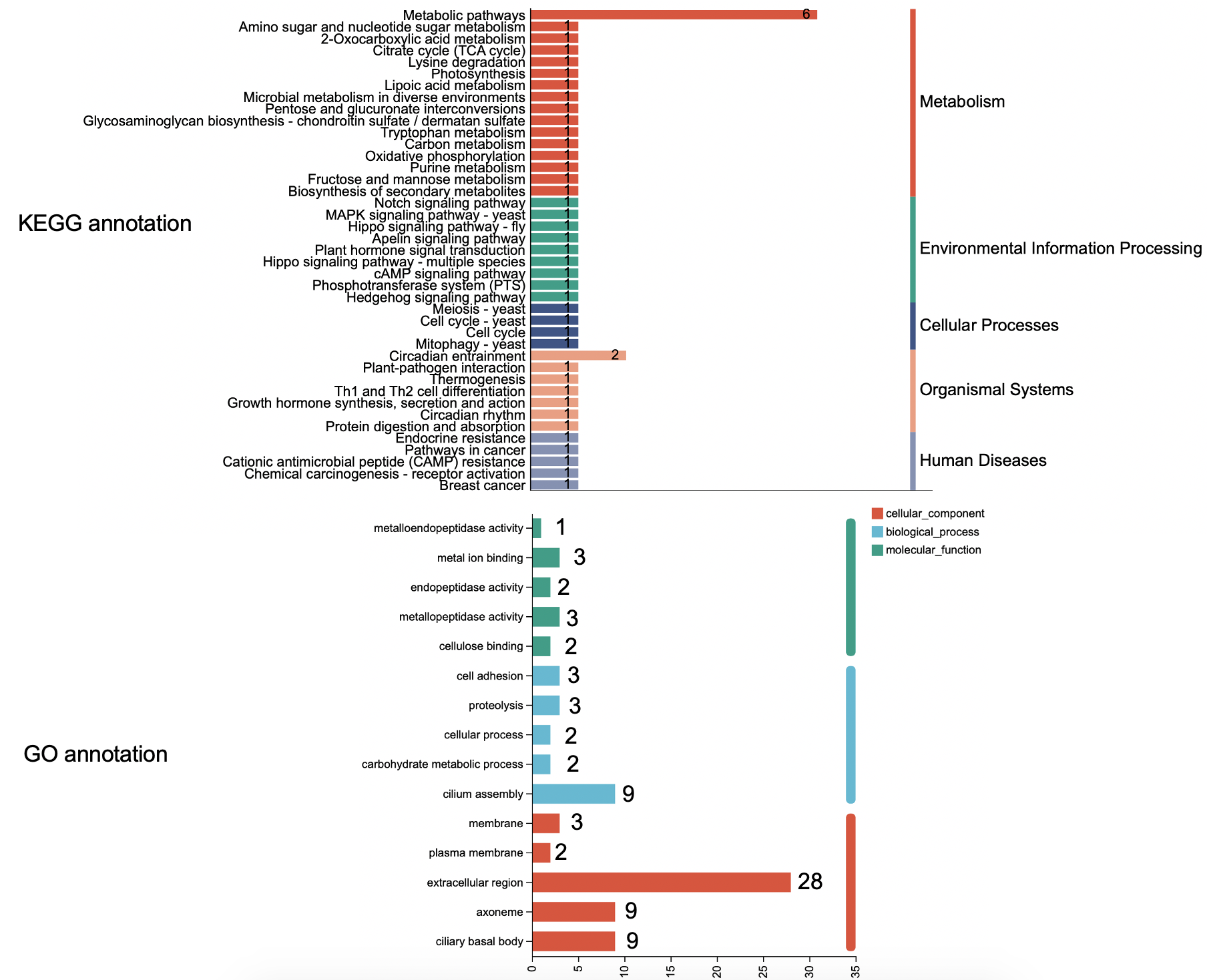


**Figure S3**. KEGG annotation and GO annotation results of brown algal PR-1 proteins.


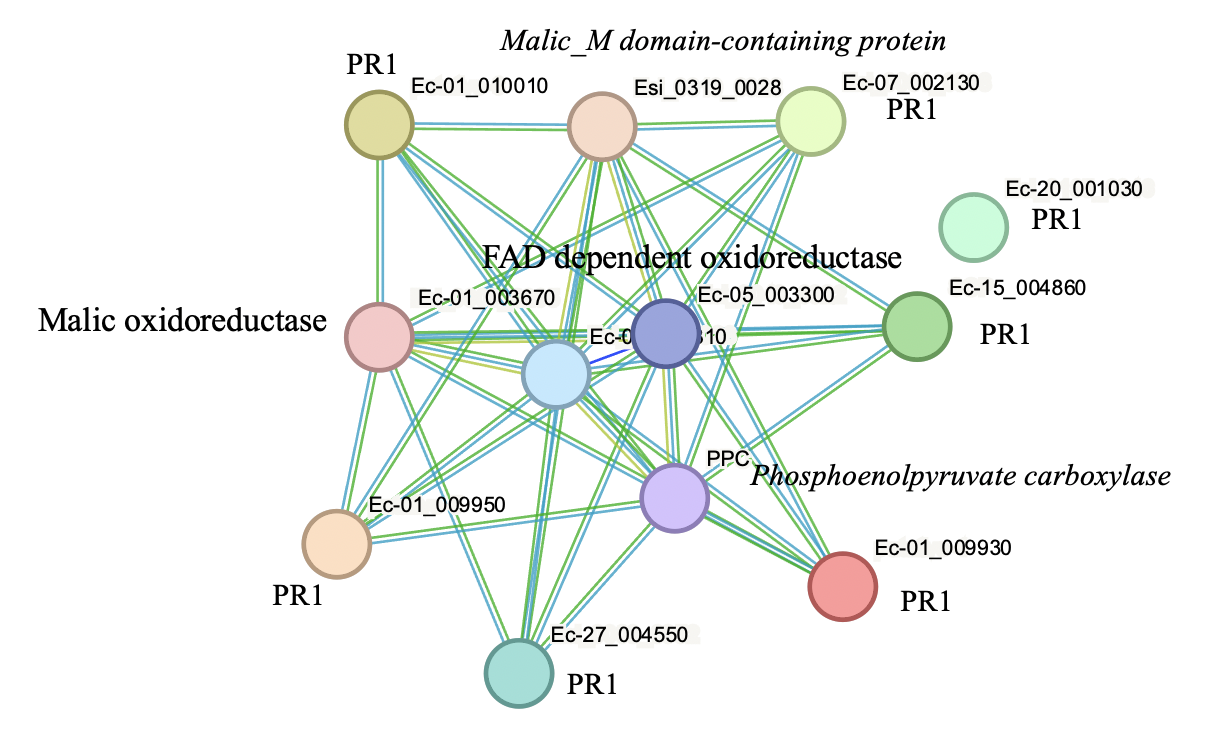


**Figure S4**. The interaction network of the EsPR-1 proteins predicted by STRING.
